# Dopamine signals encode internally determined subjective value regardless of externally indicated reward attributes

**DOI:** 10.1101/2023.01.20.524896

**Published:** 2023-01-20

**Authors:** Daniel F. Hill, Robert W. Hickman, Alaa Al-Mohammad, Arkadiusz Stasiak, Wolfram Schultz

**Affiliations:** Department of Physiology, Development and Neuroscience, University of Cambridge, Cambridge CB2 3DY, United Kingdom

**Keywords:** BDM, second-price auction, bidding, ranking, incentive compatible

## Abstract

The dopamine reward prediction error signal is known to be subjective but has so far only been related to explicit external stimuli and rewards. However, personal choices are based on private internal values of the rewards at stake. Without indications of an agent’s private internal value, we do not know whether dopamine neurons, or any reward neurons, encode the internal value. The well-established Becker-DeGroot-Marschak (BDM) auction-like mechanism allows participants to place bids for freely stating their private internal value for a good. BDM bids are known to reflect the agent’s true internal valuation, as inaccurate bidding results in suboptimal reward (‘incentive compatibility’). In our experiment rhesus monkeys placed BDM bids for juice rewards without specific external constraints. Their bids for physically identical rewards varied trial by trial and increased overall for larger rewards. Responses of midbrain dopamine neurons followed the trial-by-trial variation of bids despite constant, explicitly predicted reward amounts; correspondingly, the dopamine responses were similar when the animal placed similar bids for different reward amounts. Support Vector Regression demonstrated accurate prediction of the animal’s bids by as few as twenty dopamine neurons, demonstrating the validity of the dopamine code for internal reward value. Thus, dopamine responses reflect the instantaneous internal subjective reward value rather than the value imposed by external stimuli.

## INTRODUCTION

‘Beauty is no quality in things themselves: it exists merely in the mind which contemplates them; and each mind perceives a different beauty’ (David Hume, 1711 – 1776).

The value of a reward is also in the eye of the beholder: it is determined by the specific benefit the reward provides for the individual agent. Thus, reward value is fundamentally subjective. However, subjective reward value cannot be directly measured; it can only be inferred from an agent’s choice; the more I prefer a reward to other rewards, the higher is its value for me. But inferring subjective value from the typically tested binary choices is very limited; an agent can choose only the higher or the lower valued option in a binary fashion.

Common behavioral methods for inferring value from choices use external stimuli to present choice options. The underlying assumption is that each presented option elicits an internal valuation process. Agents then behave ‘as if’ they choose the option that has the highest internal value for them. The external stimuli are known to elicit neuronal signals for subjectively weighted reward amounts, called utility (Stauffer et al. 2014), but we do not know whether these neuronal signals simply reflect the external stimulus indicating the subjective reward value or represent the internal subjective value elicited by the stimulus. Neuronal signals for internal processes exist in human sensory association cortex where spontaneous musical tunes and imagined speech are associated with neuronal activities that can drive melodies on pianos and speech on synthesizers (Aflalo et al. 2022; Wandelt et al. 2022). The suggestion of internal signals in the absence of outside sensory events or motor acts prompts the question whether reward neurons may carry analogous signals for internal subjective reward value distinct from externally imposed reward information.

While spontaneous tunes and imagined speech are intuitively straightforward to capture, the detection of internal representations of reward value requires specific elicitation mechanisms. The Becker-DeGroot-Marschak second-price auction-like mechanism (BDM) provides an experimentally minimal approach for revealing internal reward value (Al-Mohammed & Schultz 2021; Becker et al. 1964). Here, the bidder states the own, private, subjective value against a randomly bidding computer opponent. Inaccurate BDM bidding results in suboptimal outcome; when bidding too low, the bidder risks losing the bid, and when bidding too high, the bidder may pay too much. Thus, the BDM bid reflects the accurate internal value (‘incentive compatibility’). As such bids are fundamentally unconstrained, they may vary from trial to trial for physically identical rewards. Thus, the BDM allows to distinguish the reward value constructed by internal subjective processes from the value imposed by external information.

The present study used the BDM to investigate neuronal coding of fundamentally unconstrained internal reward value. We tested dopamine neurons whose reward prediction error signal provides a reliable readout of externally imposed but nevertheless subjective reward value (Stauffer et al. 2014). We then distinguished between internal reward value and externally imposed value by correlating the dopamine responses with BDM bids as opposed to value defined by externally predicted reward amounts. We found that the dopamine signal followed trial-by-trial variations of BDM bids but was similar with same bids for different predicted reward amounts, thus coding internally generated rather than externally imposed reward value. Correspondingly, the decoded dopamine signal predicted BDM bids with high accuracy.

## RESULTS

### Monkeys’ bids reflect internally generated instantaneous subjective values

**Figure 1.**
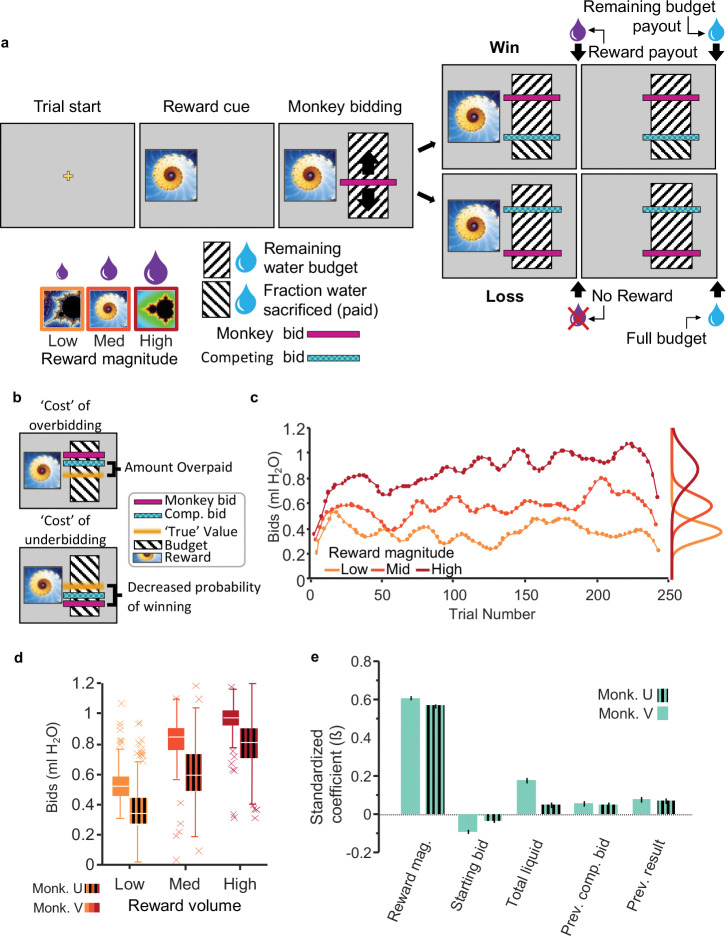
Monkeys bid *as if* they are optimizing internal subjective value. a, Becker-DeGroot-Marschak auction-like bidding task. Monkeys were trained to associate fractal images with varying quantities of juice reward. Using a lever, they bid to indicate how much of their water endowment (refilled on each trial) they would be willing to ‘pay’ for a given juice reward (willingness-to-pay; cyan bar). The ‘budget’ (hashed area) was fixed at 1.2 ml of water. After bidding, the competing bid was displayed (cyan bar), and then rewards were paid out in sequence (i.e., juice delivery, 1.5 s delay, water delivery). b, The optimal bidding strategy is to bid one’s ‘true’ value, which avoids overbidding (overpaying from the water endowment) and underbidding (less likely winning). c, Example monkey bids from a single experimental session. Monkey bids were typically normally distributed and varied coherently within and between experimental sessions. d, Average bids for all sessions. Bids are rank-ordered on average. Variance could largely be attributed to coherent changes in value over time (see figure S2). e, Mixed effects model illustrating predictors of monkey bidding. Relevant task variables were identified using a lasso model (see figure S2). A mixed-effects model was then used to determine their relative contributions to bidding independent of trial progression and between-day variability (see Methods).

We trained two rhesus monkeys to bid for fixed volumes of juice reward against a computer opponent in the BDM task (figure 1a). The task contained a sequence of events (figures 1a, S1), the most important of which was the onset of the three fractal stimuli that defined the respective three magnitudes of juice on offer. Following a task-initiation screen (trial start), monkeys were shown one of three fractal images representing three different juice volumes (Monkey U: 0.2 ml, 0.45 ml, and 0.7 ml; Monkey V: 0.3 ml, 1.0 ml, and 1.7 ml). After the fractal image, the animal was shown a ‘bid space’ representing 1.2 ml water (figure 1a; hashed black and white fill). Forward and backward movement of a lever (right hand side) resulted in the upward and downward movement of the cursor that indicated the monkey’s bid (represented by a magenta bar; figure 1a). Once the animal’s bid stayed stable for 500 ms, the computer bid was shown (green bar; randomly sampled from a uniform distribution). If the monkey’s bid was equal to or exceeded the computer bid (win), the animal received the corresponding juice reward and the remainder of the water after subtracting a water amount that corresponded to the computer bid (indicated by reversed direction of the hash fill in figure 1a). Thus, the green bar also served as cue for the water payout on win trials. If the monkey’s bid was lower than the computer bid (loss), the animal received the full water budget (1.2 ml) but no juice.

Three fractal cues for three reward magnitudes were trained extensively (>20,000 trials). The fractals for the respective juice magnitudes were displayed in pseudo-random order to generate prediction errors relative to the mean experienced reward magnitude. Monkeys’ individual bids were consistently rank ordered, and their means correlated well with juice volume (R^2^ = 0.61, p < 0.05; session average R^2^ = 0.46, p < 0.05 in 96.9% of sessions, Spearman rank correlation) (figure 1c, d). The bids fluctuated from trial-to-trial within experimental sessions (figure 1c) and from day-to-day between sessions (figure S2a). Importantly, if these fluctuations were driven by internal changes in subjective value, bid variability over time should be consistent at all three reward levels. In other words, bid fluctuations should be similar across the three reward magnitudes. We tested this possibility by analyzing the coherence of bids across reward levels and found that bid fluctuations were indeed coherent from trial-to-trial and from day-to-day (figure S2b, c; see Table S1 for summary statistics). Thus, bid variability resulted from changes in internally generated subjective values.

To identify the most critical variables for the bidding behavior, we fitted a Lasso regression model using all 29 variables (see Methods). To avoid overfitting a regression with a maximum of possible variables, the Lasso regression eliminated regressors with low explanatory power (as defined by the lambda coefficient being one standard error above the mean squared error; figure S2d). A total of seven regressor survived the Lasso elimination and was entered into a mixed effects model (Eq. 1). We eliminated the influence of trial number and session number by including them only as random effects grouping variables for the intercept. The remaining factors that significantly affected the animal’s bids included reward magnitude, starting bid, total liquid consumed, previous computer bid for the same reward magnitude, and previous bidding result (win/lose) for the same reward magnitude (figure 1e; adjusted R^2^_V_ = 0.50, R^2^_U_ = 0.41). To better understand how each regressor contributed to the monkeys’ bids independently of reward magnitude, we eliminated the influence of reward magnitude by including it as a random effect grouping variable for the intercept. The resulting modified mixed effects model identified how the following four key variables that affected bidding: starting bid, total liquid consumed, previous computer bid for the same reward magnitude, and previous bidding result (win/lose) for the same reward magnitude (figure S2e). Starting bid and total liquid consumed affected bidding more with Monkey V than Monkey U, suggesting that different features of the task uniquely contributed to individual monkey’s subjective value estimates (Monkey V: starting bid ß = −0.1 and total liquid ß = 0.18; Monkey U: ß = −0.04 and 0.47, respectively).

### Dopamine signal reflects trial-by-trial changes in subjective value

We recorded single-unit activity in the midbrain during performance of the BDM task. Neurons with wide waveforms (> 1.8 ms) and low baseline impulse rates (< 10 Hz) that responded significantly to at least one task event (p < 0.05; Wilcoxon test) were categorized as putative dopamine neurons (n = 145 for Monkey V and n = 123 for Monkey U; n = number of neurons); all other neurons were categorized as putative non-dopamine neurons (n = 114 for Monkey V and n = 113 for Monkey U).

**Figure 2.**
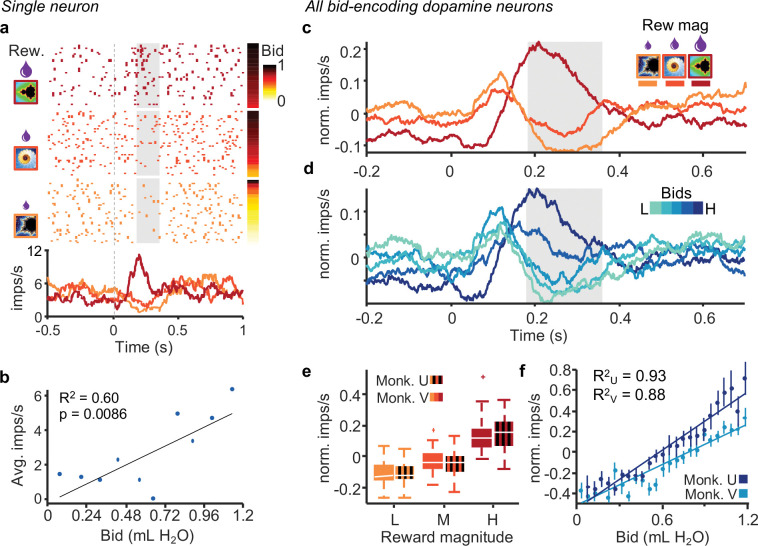
Dopamine responses reflect subjective value on a trial-by-trial basis. a, Peri-event raster and average impulse rate traces for a single dopamine neuron aligned to onset of fractal display. The raster is sorted by reward magnitude level (left) and monkey bid (right). b, Correlation of average impulse rates per bin for monkeys’ bids binned by tenths. c, Average traces of impulse rates of all bid-correlated dopamine neurons grouped by reward magnitude (Monkey V, n = 41 neurons). d, Average traces of activity of all bid-correlated dopamine neurons grouped by bid quintiles (same neurons as in panel c). e, Average normalized impulse rates during the post-event time period shown by the gray box in panels c and d. f, Mean normalized impulse rates (bids split into 25 bins; monkey U: R^2^ = 0.93, p = 4.1 × 10^−14^; monkey V: R^2^ = 0.88, p = 6.6 × 10^−12^).

Roughly one-half to two-thirds of all dopamine neurons exhibited graded responses to the external reward cues (either the fractal indicating juice amount or the cue for water payout on win trials i.e. green bar; n = 80, 65 % for Monkey V and n = 68, 47 % for Monkey U; p < 0.05, two separate single linear regressions). These dopamine responses reflected higher-order reward prediction errors at the presentation of reward magnitude-predicting stimuli (figure 1a), as observed in previous studies (Lak et al. 2014; Stauffer et al. 2014). Importantly, responses in a subset of these neurons correlated significantly with the monkeys’ bids (n = 41 for Monkey V, and n = 32 for Monkey U; p < 0.05; single linear regression). The example neuron in figure 2a, b exhibited increased activity with increase of both reward magnitude and bid in response to onset of the fractal indicating the juice amount.

We then selected dopamine neurons for their significant response to the bids, using the regression of Eq. 3. These bid-encoding responses varied also with reward magnitude well (figure 2c). The population responses of these bid-encoding neurons are shown in figure 2d. The significant relationship of the bid-encoding dopamine neurons was also seen in both animals in the normalized population responses with reward magnitude (figure 2e; Monkey U: p = 3.5 × 10^−12^; Monkey V: p = 5.6 × 10^−15^; Kruskal-Wallis Test) and with the bids (figure 2f; Monkey U: R^2^ = 0.93, p = 4.1 × 10^−14^; Monkey V: R^2^ = 0.88, p = 6.6 × 10^−12^; Eq. 3). These relationships were also seen in all dopamine neurons (figure S3). Neuronal population responses of the bid-encoding dopamine neurons varied significantly with bids for both monkeys. The same data for the population of all recorded dopamine neurons are shown in figure S3. The average responses from bid quintiles are shown for individual bid-encoding dopamine neurons in figure S4 (n = 32, Monkey U; n = 41, Monkey V) and for all dopamine neurons in figure S5 (n = 123, Monkey U; n = 145, Monkey V).

The bidding required an arm movement and was therefore correlated with movement amplitude, velocity and absement (movement amplitude × time). As dopamine responses are only very mildly modulated by movement compared to reward prediction error magnitude (Ljungberg et al. 1992; Satoh et al. 2003), our analysis focused on the bids. Indeed, testing for movement parameters in analogy to Eq. 3, we found only few dopamine neurons whose responses varied with movement velocity (1 and 10 of 123 and 145 dopamine neurons in Monkeys U and V, respectively), unsigned velocity (8 and 2 neurons), absement (2 and 5 neurons), or unsigned absement (3 and 5 neurons). Of the specifically bid-encoding dopamine neurons, even fewer neurons varied with movement velocity (only 0 and 3 of 32 and 41 neurons in Monkeys U and V, respectively), unsigned velocity (2 and 2 neurons), absement (0 and 1 neuron), or unsigned absement (1 and 2 neurons). All of these neuron numbers failed to exceed the 5% chance level. Thus, movement parameters failed to explain bid-encoding in dopamine neurons.

### Dopamine neurons reflect subjective value (bids) irrespective of reward magnitude

#### Graded coding of subjective value despite same reward magnitude.

**Figure 3.**
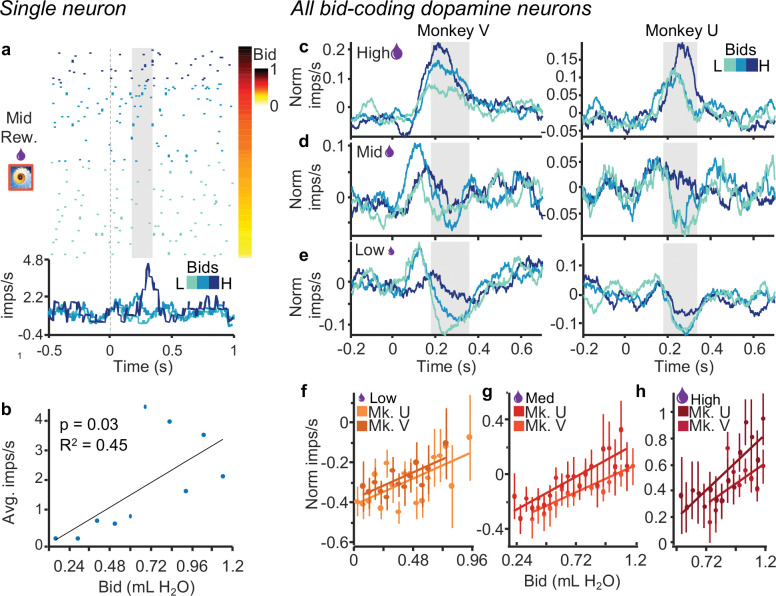
Dopamine neurons exhibit graded responses that reflect the animal’s bids irrespective of reward magnitude. a, Raster and peri-event average impulse rate for the middle reward magnitude only. The raster is sorted by bid (right) and the three shades of blue show the responses for thirds of the bid space (traces and raster). b, Correlation with average impulse rates per bin for bids binned by tenths. c-e, Traces showing normalized impulse rates for low, middle, and high reward magnitudes individually; blue shades indicate thirds of the bid space as in a. f-h, Regressions of normalized impulse rates on bids for each reward magnitude. Monkey U: Low: R^2^ = 0.56, p < 0.01; Med: R^2^ = 0.74, p < 0.001; High R^2^ = 0.67, p < 0.001. Monkey V: Low: R^2^ = 0.56, p < 0.01; Med: R^2^ = 0.74, p < 0.001; High R^2^ = 0.67, p = 0.08.

In this experiment, the bid reports the internal subjective value of the reward magnitude, and therefore the two variables are intercorrelated. Consequently, variability amongst bids for a given specific reward magnitude is solely contingent on changes in subjective value over time. Our aim was to understand whether and how dopamine neurons encode these subtle changes in subjective value. The response of the dopamine neuron shown in figure 3a, b varied significantly with the bids when only the fractal for the single middle reward magnitude was displayed (p < 0.03; single linear regression). Similar monotonic bid coding despite constant reward magnitude was seen with each of the three reward magnitudes and in both animals (figure 3c–h; Monkey V: R^2^_high_ = 0.32, p_high_ = 0.08, R^2^_mid_ = 0.83, p_mid_ < 0.001, R^2^_low_ = 0.41, p_low_ < 0.01; Monkey U: R^2^_high_ = 0.67, p_high_ < 0.001, R^2^_mid_ = 0.74, p_mid_ < 0.001, R^2^_low_ = 0.56, p_low_ < 0.01 for high, middle, and low bids, with n = 41 and n = 32 neurons, respectively). Traces for individual neurons are shown for each reward magnitude split by bid-tercile in figure S6. Thus, the dopamine neurons seemed to encode the subjective value, as indicated by bidding behavior. Because higher reward magnitude elicited higher bids (figure 1d, e), we next asked whether the bid-sensitive neuronal responses might also reflect reward magnitude.

#### Same subjective value responses despite different reward magnitudes.

**Figure 4.**
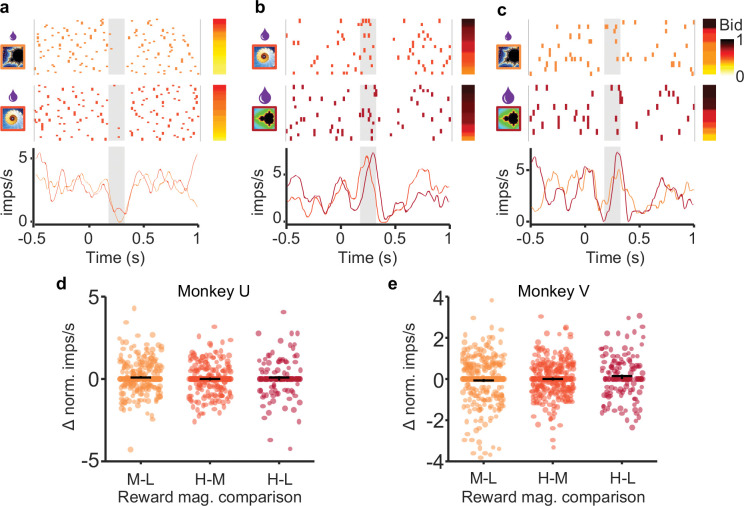
Dopamine responses exhibit similar responses with similar bids despite different reward magnitudes. a-c, Raster plots and peri-event averages for similar bids for low, middle, and high reward magnitudes, respectively. Rasters are sorted by bid (bar to the right of rasters); different shades represent lower vs. higher reward magnitudes for each comparison (left of rasters). d and e, Difference measure of normalized responses for each comparison for Monkey U (d) and Monkey V (e). No significant differences with any comparison (p > 0.05).

Above we demonstrated that dopamine neurons encode subjective value (bids) prior to the bid being made. However, bid coding does not preclude reward magnitude coding in and of itself. To test whether reward magnitude was encoded independent of bidding, we examined the responses of neurons when bids were similar for two different reward magnitudes. Because there were low numbers of perfectly matched bids between reward levels, bids within 5% of one another were compared. Comparisons with significantly different bid distributions were eliminated (see methods for complete explanation). For these similar bids (see methods), we found no difference in dopamine responses between fractals indicating small vs. medium reward magnitudes (figure 4a), medium vs. large reward magnitudes (b), and small vs. large reward magnitudes (c). Instead, the dopamine responses reflected the bids the animal made rather than the reward magnitude indicated by the fractals.

We next tested response differences for similar bids across *all* bid-encoding neurons as a group. For similar bids (< 5%), we subtracted responses to lower reward magnitudes from responses to higher reward magnitudes; for this test, computed differences greater or lesser than zero indicate responses driven by reward magnitude. Concurrently, we found no difference in responses between higher and lower reward levels for similar bids (p > 0.05 for each comparison; Wilcoxon signed rank test) (figure 4d, e). Together, these data suggest that the dopamine neurons encoded the internally generated subjective value as expressed by the bid and not the reward magnitude indicated by the fractals.

### Dopamine responses decode future bids

Given that the dopamine response to the fractal stimuli reflected the animal’s bid rather than the reward magnitude indicated by the fractal, the question arose whether this neuronal response could decode the bid the animal was going to make a few seconds later. We addressed the question by using a Support Vector Regression (SVR) that can decode on a continuous scale, rather than binary distinctions typical for standard Support Vector Machine (SVM) classifiers. As the data derived from several weeks of recording, their non-simultaneous nature provided a rather conservative estimate of the decoding capacity of the dopamine response. We trained the SVR on neuronal responses from 80% of the bids, randomly selecting responses in each neuron from each tenth of the bid space. Using the remaining 20% of the bids and neuronal responses, we then tested the accuracy with which the model predicted the monkeys’ bids, using 150 iterations of 100 randomly selected trials (see Methods).

**Figure 5.**
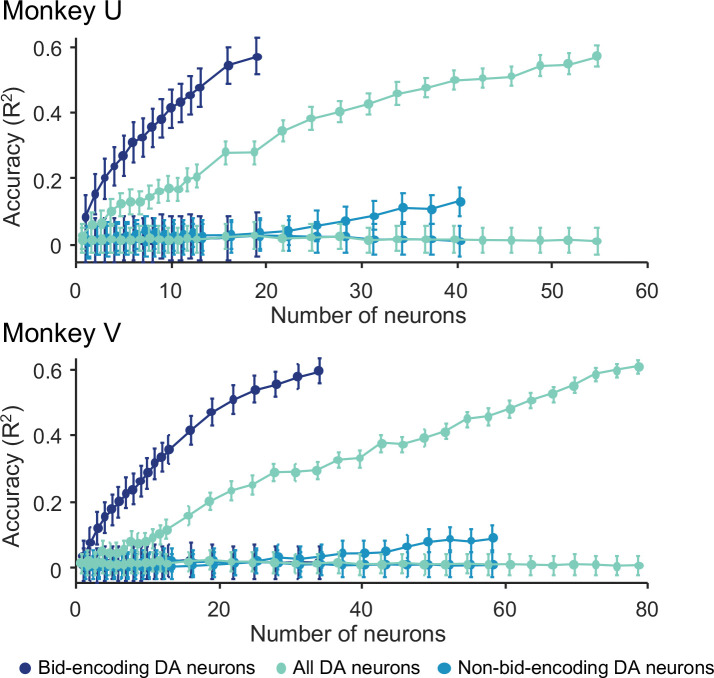
Results from analysis by Support Vector Regression (SVR) for the two monkeys. The model was trained on responses and bids using 80% of the data. Bids were predicted from responses with the remaining 20% of the data. Bid prediction accuracy is shown as R^2^ (ordinate) for bid-encoding dopamine neurons (dark blue; DA), all dopamine neurons (second curve from top, light cyan), and non-bid-encoding dopamine neurons (light blue), and shuffled data (three flat curves at bottom in corresponding colors). Neurons were added to the model in random order (see Methods).

When we added randomly selected responses from randomly selected bid-encoding dopamine neurons to the model, we found that decoding accuracy was low for single neurons but quickly increased to about 60% with approximately 20 neurons (figure 5, dark blue). The accuracy was lower when we included all other dopamine neurons (light cyan) and was lowest with only the non-bid-encoding neurons (light blue), suggesting that the internal subjective value was largely encoded in the population of bid-encoding dopamine neurons.

While these data show the contribution of the ‘typical’ dopamine neuron, we sought to assess the upper limit of decoding accuracy of dopamine neurons. For this aim, we added neurons to the model from best-encoding to worst-encoding, ordered by explained variance (R^2^ of individual bid-encoding dopamine neurons (Eq. 3). We found that decoding accuracy reached asymptote with relatively few neurons in both animals (figure S7a, b), suggesting high-fidelity encoding of internal subjective value in even smaller groups of dopamine neurons. The decoding accuracy did not improve by combining these neurons with non-bid-encoding neurons (light cyan in figure S7a, b). As anticipated, when we added bid-encoding dopamine neurons in the reverse order, from the worst-encoding to the best-encoding, decoding accuracy fell below that of the average dopamine neurons (figure S7c, d), suggesting that most of the decoding accuracy was derived from the best bid-encoding neurons and that little was gained from the worst bid-encoding neurons (the last neurons in figure S7c and d were the first neurons in panels a and b, respectively).

In conclusion, the SVR demonstrated limited fidelity of coding in single neurons that improved rapidly with small populations. The high accuracy is remarkable given that decoding continuous behavior, such as bidding in the BDM, is more challenging than traditional classification of binary choices.

## DISCUSSION

These data show that the phasic dopamine reward signal encodes subjective value that is internally generated by the brain from externally presented information (stimuli, rewards). We used the BDM auction-like mechanism to estimate the internal reward value in an accurate manner (incentive compatibility) without the value being dictated by external reward information (figure 1). We found that the reward responses of dopamine neurons followed the animal’s BDM bids (figure 2), both with constant and with varying reward amounts (figures 3, 4). The SVR decoder predicted the BDM bids accurately from the dopamine responses, which demonstrates the validity of the neuronal code for internal reward value (figure 5). Thus, the dopamine responses encoded internal subjective reward value rather than the value imposed by external reward information.

The BDM constitutes a key estimation mechanism for internal reward value by encouraging subjects to truthfully report their internal subjective value, a property called incentive compatibility. The second-price nature of BDM prevents incorrect bidding; an exaggerated bid would allow the price to rise beyond the bidder’s own value, whereas an understated bid incurs the risk of losing out on the desired good. In this way, BDM bids reveal the true internal subjective reward value without requiring one to infer reward value from observable choice, as often used in standard neurophysiological experiments. Thus, BDM bids are adequate without being constrained by specific option sets with externally imposed value, and there is no instance where the subject is forced to choose between options, eliminating the potential confound of stimulus-defined value for choice options (Al-Mohammad & Schultz 2022).

Our behavioral results correspond to the BDM characteristics of internal and subjective value estimation. The variation of BDM bids within and between sessions for the same reward magnitude demonstrated the subjective nature of the internal valuation (figure S2a). While being subjective, the animals’ bidding was meaningful, as evidenced both by the overall larger bids for higher reward magnitudes and the coherence of bids among magnitude levels (figures 1c–e, S2b, c). The subjective nature of BDM valuation was also apparent in inter-individual valuation differences, as satiety varied substantially between the two monkeys (figure S2e; total liquid consumed). Together, these results confirmed that the animals’ bids in our BDM reflected the internal subjective value for the tested juice volumes generated by the brain from external reward information.

The reported dopamine reward prediction error responses to the occurrence of the fractal stimuli seemed to follow both the BDM bids and the externally indicated reward amounts (figure 2). However, the dopamine responses varied with the bids even when reward amounts were held constant (figure 3), and the responses were similar with similar bids for different reward amounts (figure 4). Thus, the dopamine responses reflected the bids and not the reward amounts, suggesting coding of internally generated subjective reward value distinct from reward magnitude.

Previous studies have shown dopamine coding of subjective reward value defined by formal utility (Stauffer et al. 2014). However, utility represents subjective value as a (usually nonlinear) mathematical function of externally indicated reward amount (Bernoulli, 1738; von Neumann and Morgenstern, 1944; Savage 1954); utility represents subjectively weighted externally indicated reward value, which contrasts with the internal subjective reward value revealed by BDM bids. Similarly, previous work reporting variations of dopamine neurons with subjective value inferred from observable behavioral choices (Morris et al. 2006; Lak et al. 2014) concerned the subjective weighting of externally indicated reward value rather than the internal subjective value assessed by BDM bids. Thus, the currently reported covariation of dopamine responses with BDM bids goes one step beyond the subjective weighting of externally imposed reward amount represented by utility functions and observable choices. Thus, the reported dopamine responses reflect internally generated subjective value of reward regardless of reward magnitude itself. The information from externally presented reward cues is apparently weighted subjectively as utility and translated into internal subjective value assessed by BDM, and the dopamine responses follow the internal subjective value revealed by BDM bids.

The SVR results demonstrate that dopamine responses to the reward cues reliably predicted the subsequent BDM bids made by the animal (figure 5). By adding randomly selected dopamine responses to the decoding model, the low decoding accuracy increased quickly to about 60% with 20 – 30 neurons. Adding neurons from best-coding to worst-coding confirmed the small neuron numbers required to accurately decode the bids (10 – 20 neurons) (figure S7a, b). As previous studies have shown that dopamine excitation drives behavior (Olds & Milner 1956; Corbett & Wise 1980; Tsai et al. 2009), our data would suggest that the dopamine response to the fractals may be instrumental for generating useful bids; only a small population of neurons (10 – 20) would be necessary for this effect. However, while the shuffled data resulted in an accuracy of 0%, the accuracy of 60% with the SVR is much smaller than the 80 – 90% accuracy achieved with binary decoders such as nearest neighbor, linear SVM and discriminant analysis classifiers (Quiroga et al. 2006; Grabenhorst et al. 2012; Chang & Tsao 2017; Pastor-Bernier et al. 2019; Koren 2021; Aflalo et al. 2022). A possible explanation for the lower accuracy of SVR as compared to a binary classifier may lie in the continuous nature of the SVR, and that accuracy is possibly further reduced due to binning the bids necessitated by our limited number of trials per bid. Thus, the observed decoding accuracy may represent a conservative estimate of the predictive capacity of the neuronal responses. While these quantitative considerations are technically important, the observed decoding of bids from dopamine responses confirms the validity of the dopamine signal for coding internal subjective reward value expressed behaviorally by the bids, which is important as the bids are made without a constraining external option set.

Taken together, these data demonstrate the ability of dopamine neurons to encode internal subjective value regardless of externally presented reward information. The BDM auction-like mechanism provided an appropriate behavioral mechanism for directly revealing the animal’s internal valuation. The SVR decoder demonstrated that the dopamine signal was capable of predicting BDM bids, thus suggesting a valid and precise neuronal code for internal value. Future studies should aim to uncover whether the observed encoding of internal reward value is unique to dopamine neurons or whether it might be a widespread feature of reward processing in the brain.

## EXPERIMENTAL PROCEDURES

### Animal ethics, welfare and surgical implantation

We used two adult male rhesus monkeys (*Macaca mulatta*; Monkey V: 11 kg and Monkey U: 17.5 kg). This research has been ethically reviewed, approved, regulated and supervised by the following UK and University of Cambridge (UCam) institutions and individuals: UK Home Office, implementing the Animals (Scientific Procedures) Act 1986, Regulations 2012, and represented by the local UK Home Office Inspector, UK Animals in Science Committee, UK National Centre for Replacement, Refinement and Reduction of Animal Experiments (NC3Rs), UCam Animal Welfare and Ethical Review Body (AWERB), UCam Biomedical Service (UBS) Certificate Holder, UCam Welfare Officer, UCam Governance and Strategy Committee, UCam Named Veterinary Surgeon (NVS), and UCam Named Animal Care and Welfare Officer (NACWO).

The two monkeys were housed in adjoining cages and placed on a restricted water regimen calibrated by body weight. Behavioral data were acquired from both animals for a prelusive publication (Al-Mohammad & Schultz 2022). Each weekday, we transported the animals to the experimental laboratory in an individually adjusted primate chair (Crist Instruments). Animals sat in this chair for the duration of the daily tests, which never exceeded 5 hours. We provided animals with fruit and vegetable enrichment on Friday evening and ad-libitum access to water throughout Friday evening and Saturday.

We implanted Monkey U with a titanium headpost (Crist instruments) used for head fixation and later implanted a recording chamber after the headpost had integrated with the bone. For Monkey V, we implanted head-fixation hardware concomitantly with the recording chamber. Chambers were centered on the skull laterally using a stereotaxic head holder and a Kopf stereotaxic manipulator. After recovery from surgery, we drilled craniotomies above recording sites and chambers were monitored and cleaned daily. Once experiments were completed, recording sites were marked with electrolytic lesions (15–20 μA, 20–60 s). Upon completion of the experiment we sacrificed the animals by administering an overdose of sodium pentobarbital (90 mg/kg, IV) and subsequently perfused with 4% formalin in 0.1 M phosphate-buffered saline. We confirmed recording positions histologically from 40 μm slices stained with cresyl violet.

### Experimental setup

During experiments, animals were head-fixed while seated in a primate chair. All experiments were performed in a dimly lit experimental isolation booth (Crist instruments) to minimize disruption. The monkeys were positioned so that their eyes were ~70 cm from a computer monitor. The joystick-lever was attached to the chair and made accessible via ~15 cm^2^ opening in the front of the chair. Water and juice were delivered through separate spouts positioned ~ 5 mm from the animal’s mouth. Fluid delivery was controlled by gravity-fed solenoid valves connected to 1 L beakers using silicone tubing. Juice and water delivery valves were calibrated to deliver precise volumes (SD < 0.01 ml). The monkeys were trained to use a custom-made touch-sensitive joystick (Biotronix Workshop, University of Cambridge) to interact with the task displayed on a computer monitor as previously described (Al-Mohammad & Schultz 2022). The joystick was only movable in the x and y directions.

### BDM elicitation of subjective value

The BDM is a second price sealed-bid auction-like mechanism that has been shown to elicit truthful estimates of internal subjective value on a single-trial basis. Typically, a BDM bidder will garner the highest payoff by bidding exactly the value they place on the good. Economists refer to this as incentive compatibility: the optimal strategy is to bid one’s true subjective value. Bidding too high (overbidding) increases the risk of overpaying, and bidding too low (underbidding) increases the risk of not obtaining the desired item (figure 1a, b) (Lusk & Shogren 2007). Three features are key to the BDM’s incentive compatibility: (1) the second-price nature is essential for revealing the true subjective value because the opponent’s bid is unknown; it prevents overbidding because the unknown bid of the opponent may exceed the internal value and thus result in overpaying; it prevents underbidding because the opponent might outbid them, and (2) the bids are hidden until all bids have been submitted (sealed bid auction). Thus, BDM is akin to a private value auction, as subjects are not able to infer the value other bidders place on the good. As opponent bids are drawn randomly from a uniform distribution, a common value cannot be surmised, even with consecutive trials. Many details of the bidding performance of the animals used in this study have been presented before (Al-Mohammad & Schultz 2022), and only the behavioral results relevant for the current neuronal analyses will be described here.

The described characteristics explain our rationale for using the BDM. Any study of internally determined reward value requires that the measured events, namely the bids given by the animal, reflect the true internal subjective value at each moment. The incentive-compatible nature of the BDM provides exactly that assurance. Further, the BDM does not require a biological opponent, which makes the experimentation less complicated and simplifies the interpretation of neuronal data by avoiding confounds from an opponent’s behavior.

Recent experiments demonstrated that rhesus monkeys can show meaningful performance in behavioral tasks implementing a BDM (Al-Mohammad & Schultz 2022). On every trial, the monkey bid against a computer opponent using a joystick; the animal paid from a water endowment that had been allocated on every trial with the same amount. If the animal’s bid equaled or exceeded the computer bid, the animal won the auction and paid the price defined by the computer bid (second-price nature of BDM); thus, the animal received the juice it had bid for, plus the rest of the water endowment after subtraction of the computer bid. If the animal’s bid was below the computer bid, it lost the auction and received the full water endowment (figure 1b). To increase the number of trials per reward magnitude, monkeys bid for only 3 reward magnitudes. The range of reward magnitudes was calibrated to each animal’s preferences so that the full range of bids were well represented. In addition, monkeys bid for fixed goods rather than for lotteries, which avoided confounds from the animal’s risk attitude. Our task also circumvented the endowment effect (a tendency to over-value previously acquired goods), as monkeys do not ‘pay’ an amount already acquired but rather indicate the amount of water they are willing to forgo from water paid out at the end of the trial. Specifically, monkeys receive a water payout on every non-error trial (see below) regardless of whether they win or lose the auction; their bids reflect how much water they are willing to forego on each trial to get the juice reward.

### BDM Task

BDM trials were initiated with a yellow cross at the center of the screen (figure 1a). After 0.5 s, a fractal representing one of three different juice volumes appeared. After 1.0 s, a vertically oriented rectangle appeared that denoted the bid space that was defined by the smallest and largest reward amount the animal could bid for (0 and 1.2 ml; hashed black lines on white background). A bid cursor overlayed the bid space rectangle (magenta). Forward and backward movement of the joystick generated up and down movements of the cursor within the bid space. Bidding had to be initiated within 0.5 s and stabilized within 5 s, otherwise the trial was terminated and the screen briefly flashed red indicating a failed trial and a wait penalty equal to the remaining trial time plus 2 seconds. Letting go of the joystick at any point during the trial or moving during any period except the bidding epoch also resulted in trial termination. The total bid space represented 1.2 ml of water; monkeys’ bids indicated how much water they were willing to sacrifice for a given fractal. Once the monkey’s bid was stable for more than 0.5 s, the bid of the computer opponent appeared and the direction of the hashed lines below the computer bid reversed, indicating the amount of water to be ‘paid’ for obtaining the juice if the animal won the BDM (second price). If the monkey won the BDM, the juice was paid out and the fractal would disappear from the screen after 1 s, followed by water pay out that lasted up to1 s (1.2 ml minus the computer bid measured in ml, which reflected the second price character of BDM) and removal of the bid space from the screen. If the monkey lost the BDM, the fractal disappeared and the water was paid out in full (1.2 ml).

### Behavioral analysis

BDM bids reflect subjective value and change from trial to trial. This can be demonstrated formally for expected-utility maximizers (see Lusk & Shogren 2007) and has been supported empirically in rhesus monkeys in previous work from our laboratory (Al-Mohammed & Schultz 2022). Here we sought to further test whether changes in bidding resulted from changes in value or from other value-irrelevant task features. To test which elements of the task contributed most to bid variance, we first used a cross-validated lasso regression (lasso function, Matlab) to identify variables that contributed to bid variability. Lambda (tuning parameter) was selected by taking the value corresponding to one standard error above the mean squared error based on 2000-fold cross-validation (figure S2d). We used the following 29 regressors in the lasso model: 1) reward value, 2) starting bid, 3) previous total liquid, 4) day of week, 5) session number, 6) previous trial failure, 7) previous trial result, 8) previous result from trial with same reward magnitude, 9) trial number, 10) competing bid t-1, 11) competing bid t-2, 12) competing bid t-3, 13) competing bid t-5, 14) competing bid t-7, 15) competing bid for the same reward magnitude t-1, 16) competing bid same reward magnitude t-2, 17) competing bid same reward magnitude t-3, 18) competing bid same reward magnitude t-4, 19) competing bid same reward magnitude t-5, 20) competing bid same reward magnitude t-6, 21) competing bid same reward magnitude t-7, 22) competing bid same reward magnitude t-8, 23) competing bid same reward magnitude t-9, 24) competing bid same reward magnitude t-10, 25) average of competing bid same reward magnitude t-2 to t-1, 26) ) average of competing bid same reward magnitude t-3 to t-1, 27) ) average of competing bid same reward magnitude t-4 to t-1, 28) ) average of competing bid same reward magnitude t-5 to t-1, 29) ) average of competing bid same reward magnitude t-6 to t-1.

Variables remaining in the correspondent model were then used in a mixed-effects model to identify which had the largest impact on bidding behavior (figure 1e).

Eq. 1 $$y=\beta_{0ij}+\beta_{1}\;RewardMagnitude_{ij}+\beta_{2}\;StartingBid{\;}_{ij}+\beta_{3}\;TotalLiquid_{ij}\;\;+\beta_{4}\;PreviousCompetingBid_{ij}+\beta_{5}\;PreviousResult_{ij}+b_{0ij}\;\;+b_{i1}\;TrialNumber_{ij}+b_{i2}SessionNumber_{ij}+\varepsilon_{ij}$$

Because reward magnitude is highly intercorrelated with the subjective value and therefore with the bid, we sought to determine what other factors, besides reward, most prominently predicted changes in bids. For this we used a reduced mixed-effects model with reward magnitude included as a random effect (figure S2e).

Eq. 2 $$y=\beta_{0ij}+\beta_{1}\;StartingBid{\;}_{ij}+\beta_{2}\;TotalLiquid_{ij}\;\;+\beta_{3}\;PreviousCompetingBid_{ij}+\beta_{4}\;PreviousResult_{ij}+b_{0ij}\;\;+b_{i1}\;RewardMagnitude_{ij}+b_{i2}\;TrialNumber_{ij}\;\;+b_{i3}\;SessionNumber_{ij}+\varepsilon_{ij}$$

If changes of individual bids reflect changes in subjective value across trial, then these changes should be apparent in bids across all three reward magnitudes. We tested this by measuring bid coherence within and between sessions. For within session coherence, bids were interpolated for each reward magnitude to create three equally populated vectors of bids to retain trial-by-trial temporal fidelity. Each vector was then correlated with the others in three separate tests comparing low reward magnitudes to mid magnitudes (L:M), mid magnitudes to high magnitudes (M:H), and low magnitudes to high magnitudes (figure S2b). The resulting rho values provide a relative estimate of coherence with values above zero indicating positive coherence. Note that because values were interpolated for trials where a given reward magnitude was not represented, this analysis can only provide a lower bound for the estimated coherence.

### Electrophysiological recording and analysis

Electrophysiological signals were recorded using electrodes made in house or ordered from Alpha-omega (125 μm diameter, 60-degree bevel). Electrodes were loaded into a sterile 23-gauge stainless steel cannula which was used to pierce the meninges and stabilized the electrode’s path through the brain. Electrodes were lowered into the midbrain using an electrode micromanipulator from Nan instruments (model: CMS) or Narishige (model: MO-97). Recordings were amplified and band-pass filtered from 100 to 5000 Hz (custom hardware and Bak Electronics). Recordings were digitized using a National Instruments data acquisition card and visualized with custom Matlab (Mathworks) software. Neuronal impulses were sorted off-line using Spike2 version 7.8 (Cambridge Electronic Design).

### Ventral midbrain localization

Coordinates for the recording sites in the ventral midbrain were determined using sagittal radiograph images of the head in a stereotaxic frame and electrophysiological signatures of surrounding cell groups. Specifically, animals were placed in a stereotaxic frame and a cannula was inserted in the center-most position of x-y plane of the recording chamber. Bone features (interaural origin and the clinoid process of the sphenoid bone) were used to determine the approximate anteroposterior and dorsoventral positions of familiar nuclei. Using these positions as anchors, stereotypical electrophysiological responses from the red nucleus and ventral posteromedial nucleus of the thalamus guided the localization of the recording sites of dopamine neurons in the ventral midbrain.

### Statistical analysis of dopamine neuron responses

Dopamine neurons were identified using canonical criteria: a wide impulse waveform (> 1.8 ms), low baseline impulse activity (< 10 Hz), and consistent responses to unpredicted reward delivery. All neuronal impulse data were binned in 1 ms bins for analyses. Population analyses were performed with all dopamine neurons; analyses of bid-encoding neurons were performed using only dopamine neurons that exhibited a positive correlation with the monkeys’ bids (see below). All statistical analyses were performed on raw activity (for single neurons) or z-normalized activity from time-windows defined by gray boxes in figures (for all bid-encoding neurons and population analyses). Average traces shown in figures were smoothed with an 80 ms or 100 ms moving average. For group-level analyses, data were z-scored to account for variance among neuronal activity. Bids were discretized into bins for individual neuron analyses and for group-level analyses to obtain more accurate estimates of average responses for a given bid-range (e.g. by splitting the overall bid range into tenths and averaging the neural responses within bins; for specifics, see Results).

Correlations between bids and neuronal responses were assessed using the following linear regression:

Eq. 3 $$y=\beta_{0}+\beta_{1}\;Monkey\;Bids$$

To test whether subjective value, as expressed by the bids, was driving changes in activity independent of reward magnitude, responses were correlated with bids when reward magnitude was held constant (figure 3a–h). This was further tested by comparing neuronal responses for matched bids between reward magnitude levels for individual sessions (figure 4). For this analysis, for each experimental session, responses corresponding to similar bids (within 5%) made for two different reward magnitudes (medium vs. low, high vs. medium, high vs. low) were pooled across all neurons and compared using a paired Wilcoxon sign-rank test. Significant differences in neuronal activity in this test would be indicative of responses driven by reward magnitude independent of bid. No difference indicates that neuronal responses were driven by bids independent of reward magnitude.

### Neural decoding with Support Vector Regression (SVR)

We used Linear SVR to predict the monkeys’ bids based on the responses of dopamine neurons to the fractals. The continuous bids were discretized into 10 non-overlapping bid ranges from [0–0.1] to [0.91–1.0]. The model was subsequently trained on neuronal responses from each bid range. Neurons with fewer than 10 trials in each bid range were excluded. For neurons with >10 trials in a bid range, 10 trials were selected at random, providing 100 randomly selected trials (figure S7). The SVR model was adapted from methods used by Glaser et al. (2020) and implemented with custom written software in Matlab 2021b using ‘fitrsvm’ and ‘predict’ functions for model training and testing.

We used three separate SVR models to assess how well dopamine neurons could predict animals’ bids. Neurons were added to these models randomly or by order of explained variance for monkeys’ bids. Neurons were added to model 1 from highest to lowest correlation, in model 2 from lowest to highest correlation, and randomly in model 3. Models one and two provide a lower and upper limit of decoding accuracy, and model 3 allows for comparison with similar analyses in previous works. Model performance was assessed with coefficient of determination R2 (explained variance). Each model was tested against shuffled data (bids and response shuffled 1,000 times). The binary differences between R2 coefficients obtained for real data against the shuffled data was verified by Wilcoxon rank-sum test (p < 0.01).

Each SVR model was trained/tested using a five-fold cross-validation (80% / 20%) method: eight trials from every bid category (8 trials × 10 categories) were used for training, and the remaining two trials from every bid category were used for testing (2 trials × 10 categories; figure S8). This procedure was repeated five times, thus providing five R^2^ estimates for each set of 100 randomly selected trials. The reported explained variance R^2^ for model-predicted versus actual bid data was calculated by averaging the R^2^ values from 150 iterations of the 100-trial random selection procedure described above.

## Supplementary Material

Supplement 1
