# Supplementary material for: Dopamine neurons encode trial-by-trial subjective reward value in an auction-like task
Source: bioRxiv. 2024 May 10:2023.01.20.524896. Originally published 2023 Jan 20. Preprint. [Version 2] doi: 10.1101/2023.01.20.524896 (PMC9882283; doi:10.1101/2023.01.20.524896)
Supplement: Supplement 1 [file NIHPP2023.01.20.524896v2-supplement-1.pdf]

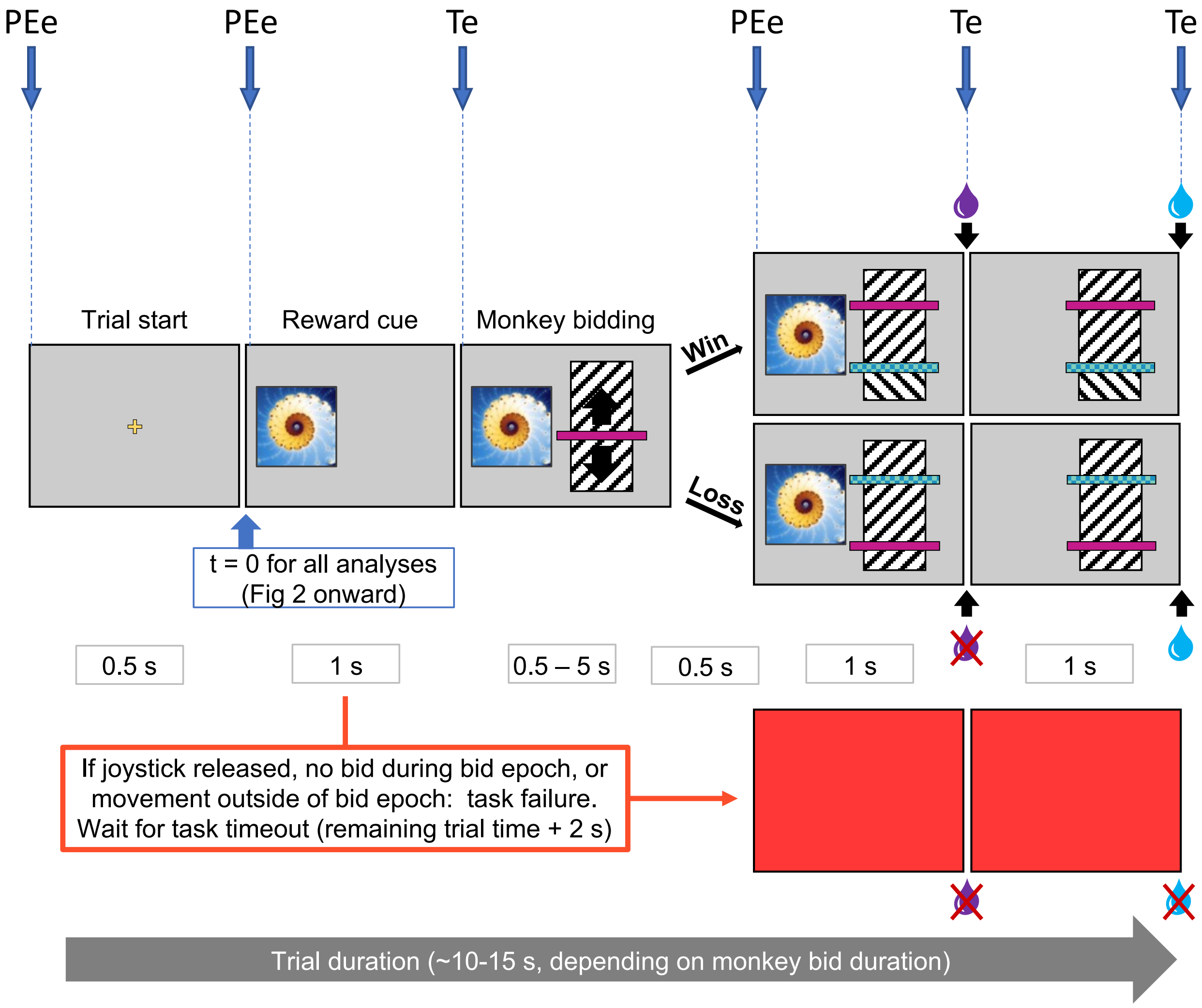

Supplementary Fig. 1. Task events. The analyzed relevant task events are shown as Te; Prediction Error events are denoted as PEe. Task events are those that may have elicited a change in neuronal response but did not constitute a prediction error because they were fully predicted. Prediction error events were those in which not all information was predicted (i.e., the monkey could not which reward magnitude will be shown next or what the competing bid would be. The fixation cross timing was slightly unpredictable due to slight timing variation between trials. It is also the earliest predictor of reward and thus would be expected to produce a large response as suggested by temporal difference models.)

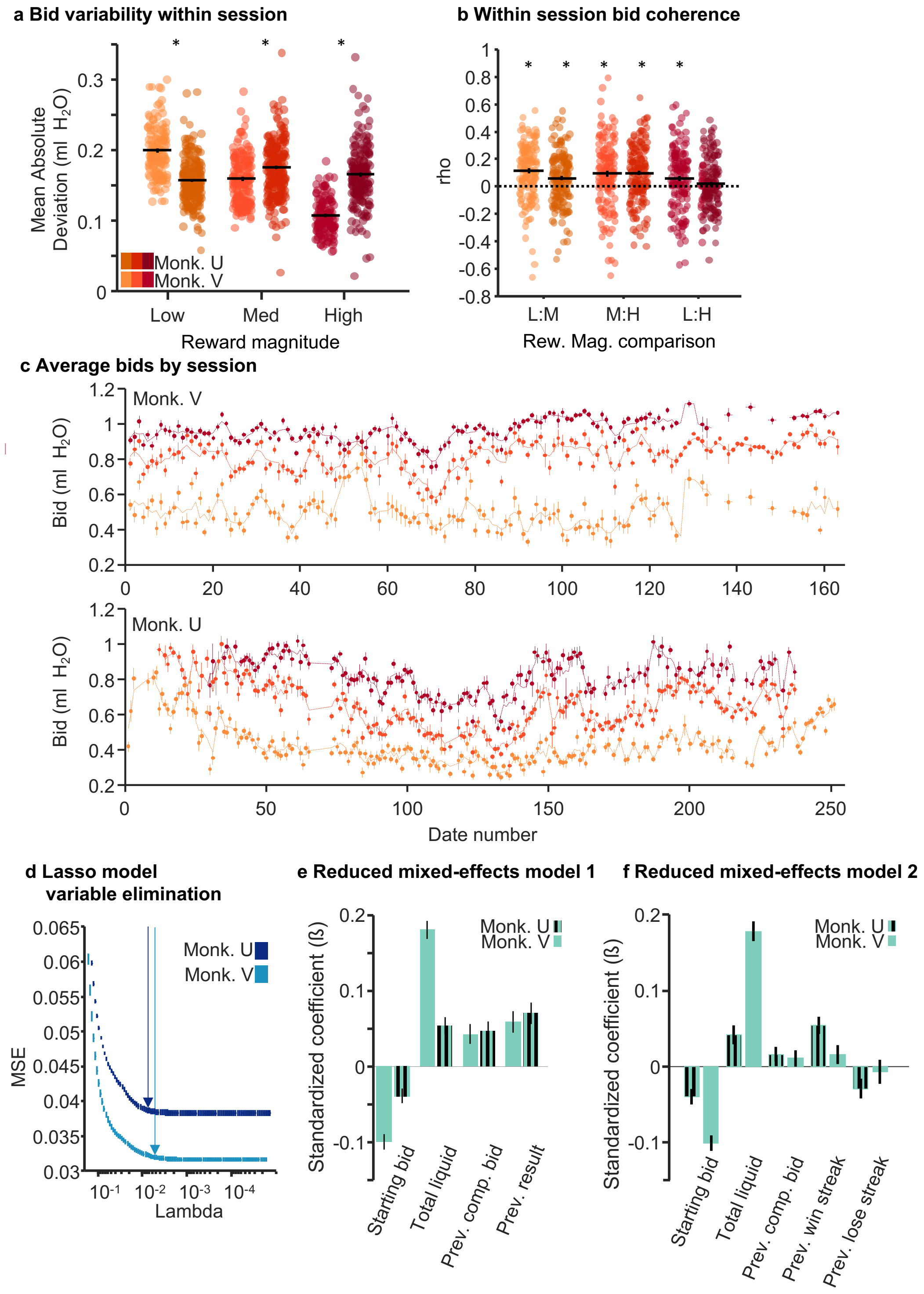

Supplementary Fig. 2. Bid characteristics. a, Bid variability within session. b, Bid coherence within session. c, Bid coherence between sessions. d, Cross-validated mean squared errors (MSE) for lasso regression model. A lasso model was used to eliminate task-irrelevant variables. The arrows indicate the lambda value that is one standard error above the minimum cross-validated error. This value was used as a cutoff; variables from this model were used in the mixed-effects model in Fig. 1 and in panel e. e, Mixed effects model controlling for reward magnitude. Mixed effects model identical to that in Fig. 1 with reward magnitude was included as a random effect. The model shows the contribution of relevant task variables to bid behavior independent of reward magnitude. f, Mixed effects model controlling for previous result and including win streak and lose streak to assess the influence of multiple consecutive wins or losses over bidding.

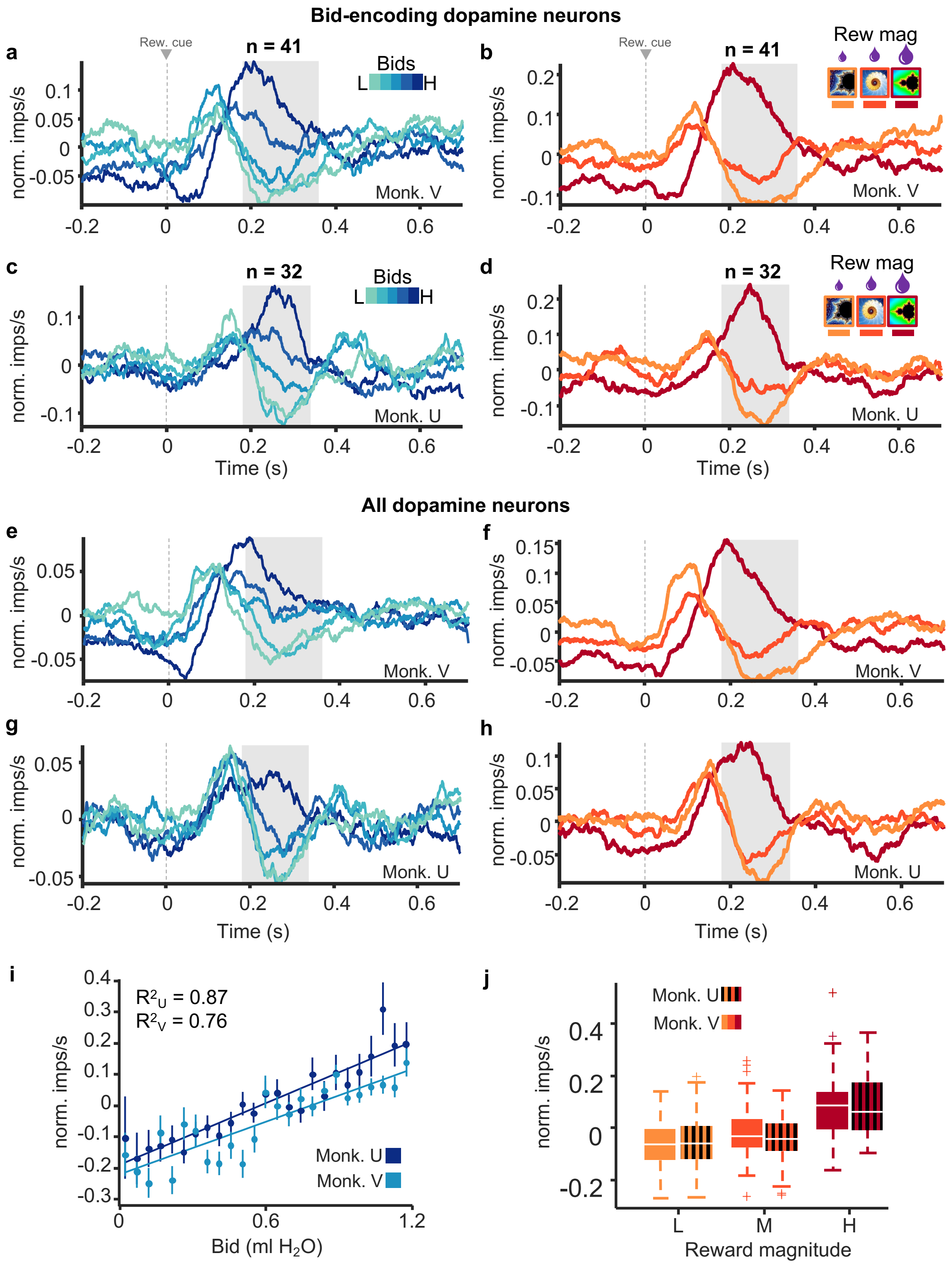

Supplementary Fig. 3. Bid-encoding and population responses. a-h, Average normalized dopamine responses for fifths of the bid-space for bid-encoding dopamine neurons (panels a-d) and for all dopamine neurons (panels e-h). i, Regression of normalized responses against bids for all dopamine neurons. Monkey U: $R^2 = 0.87$, $p = 1.03 \times 10^{-11}$, n = 32 neurons; Monkey V: $R^2 = 0.75$, $p = 1.64 \times 10^{-8}$, n = 41 neurons. j, Responses for all dopamine neurons grouped by reward magnitude. Kruskal-Wallis Test; Monkey U: $\chi^2 = 85.63$, $p = 2.55 \times 10^{-19}$, n = 123 neurons; Monkey V: $\chi^2 = 70.42$, $p = 5.11 \times 10^{-16}$, n = 145 neurons. Note that all analyses concerned only the second, value dopamine response component (gray analysis windows in panels a-h), whereas the preceding first, attentional response component varied only inconsistently.

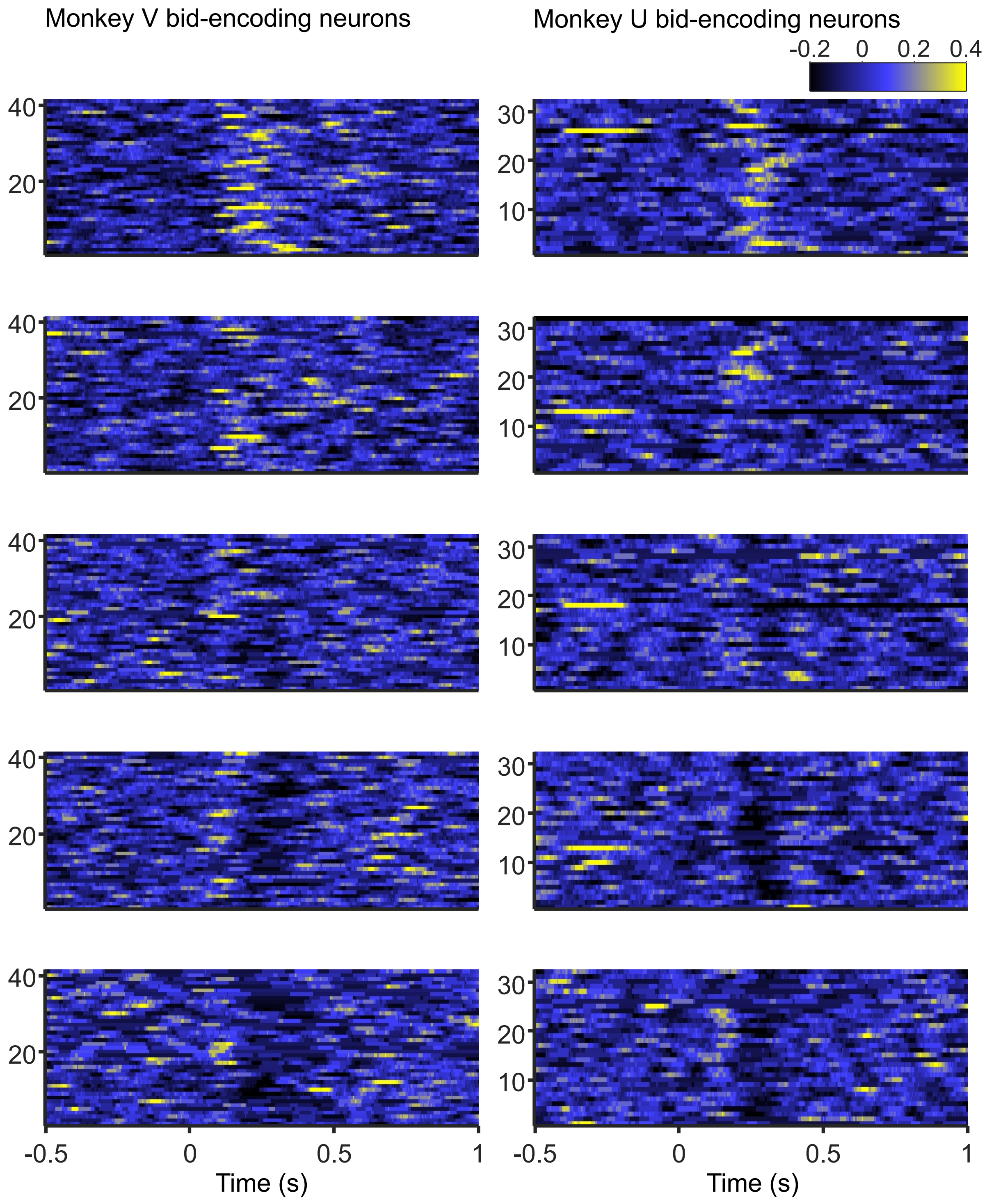

Supplementary Fig. 4. Average traces for each bid-encoding neuron for fifths of the bid-space. Average responses from the lowest fifth are shown at the bottom to the highest fifth at the top. Monkey U is on the left (n = 32), Monkey V is on the right (n = 41).

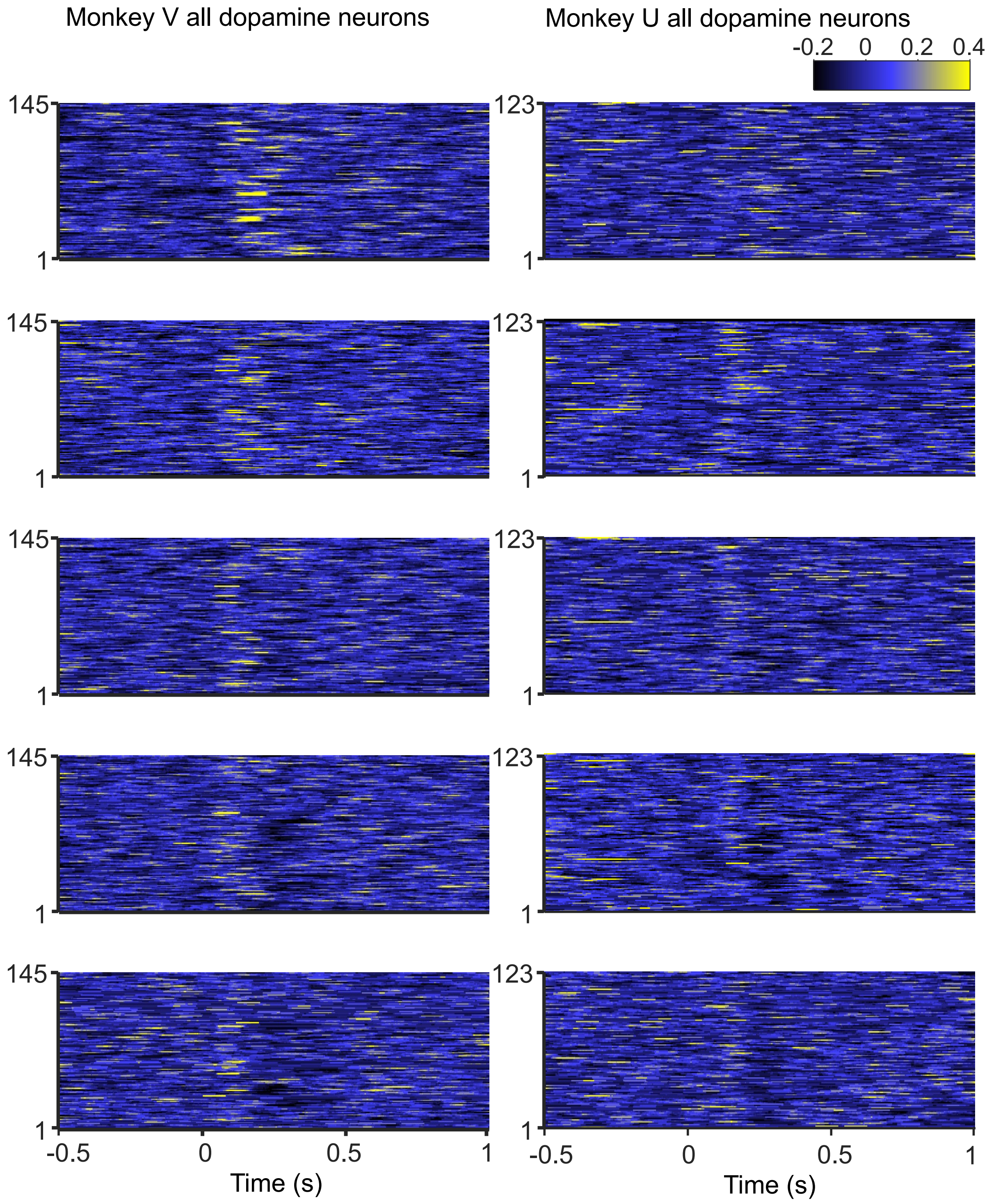

Supplementary Fig. 5. Average traces for all dopamine neurons for all five fifths of the bid-space. Average responses from the lowest fifth are shown at the bottom to the highest fifth at the top. Monkey U is on the left (n = 123 neurons), Monkey V is on the right (n = 145).

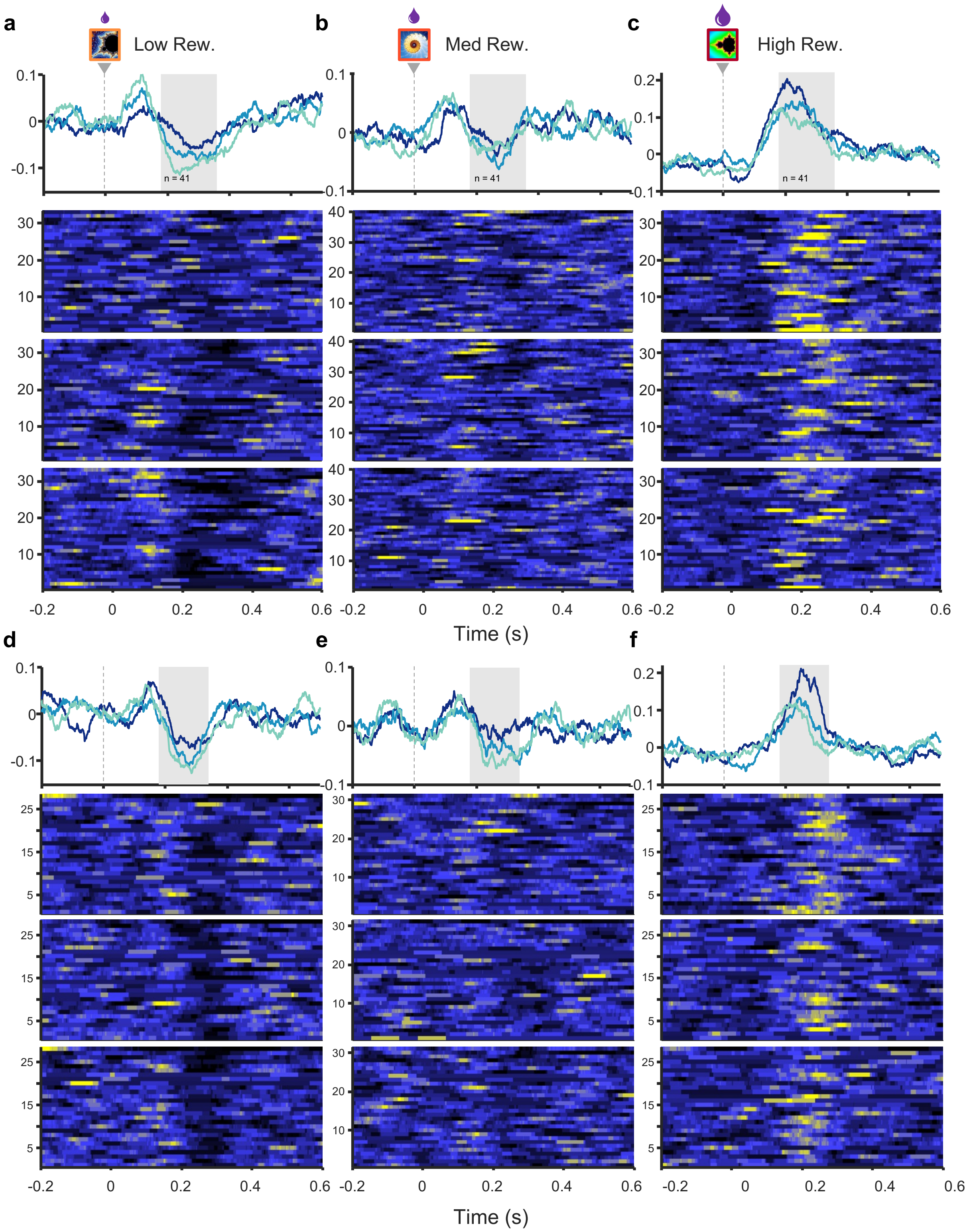

Supplementary Fig. 6. Average traces for each bid-encoding dopamine neuron for thirds of the bid-space within each reward magnitude (a, low; b, mid; c, high) for Monkey U (d-e; n = 32) and Monkey V (a-c; n = 41) . In each panel, responses from the highest, middle and lowest third are shown from top to bottom within each reward magnitude.

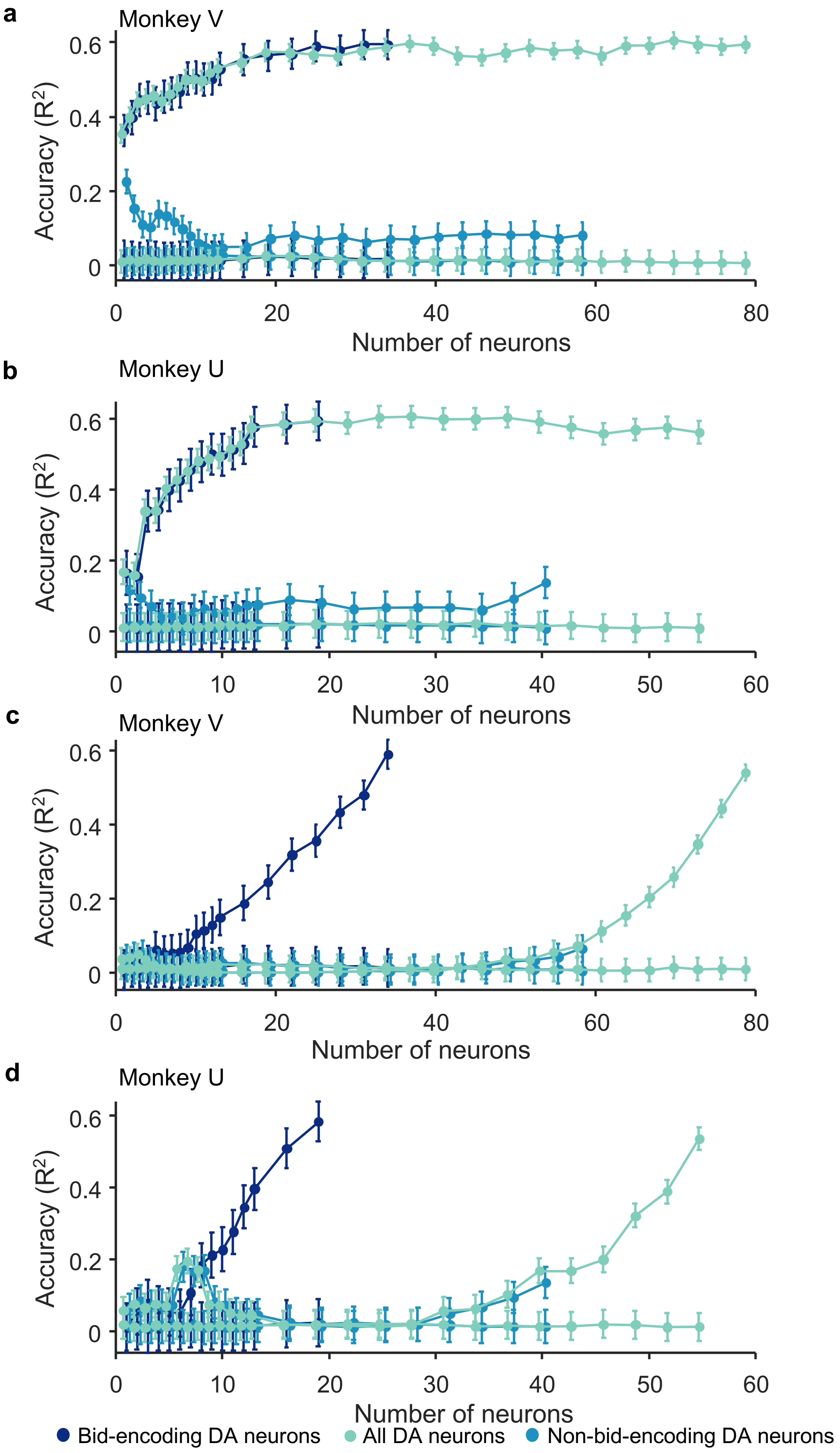

Supplementary Fig. 7. Results from analysis by Support Vector Regression (SVR) for the two monkeys. In contrast to Fig. 5, we added neurons from highest to lowest explained variance (derived from regression of individual neuron responses on bids) (a, b) and in reverse order (lowest to highest; c and d).

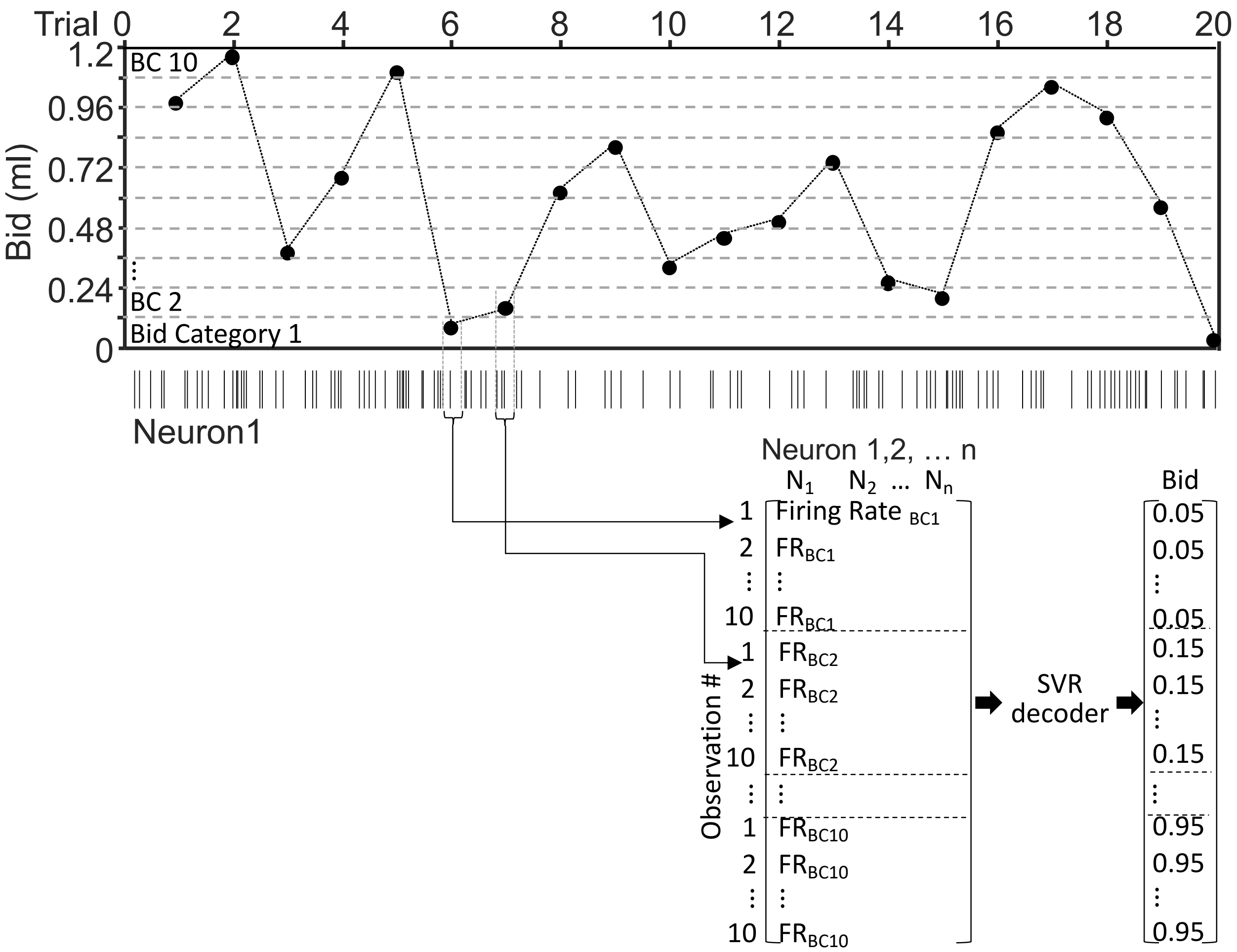

Supplementary Fig. 8. Design of the Support Vector Regression (SVR) model. Responses from 10 bid categories (BC; bid-space split into tenths) were added to the analysis such that each category was equally sampled. The bid-space was split into tenths to increase the sampling per bid category. The SVR was trained on these responses (see Methods).

Supplementary Table 1: Rho values and p values for time series bid 'coherence' (Pearson correlation).

Trial-to-trial bid 'coherence'						
	Low : Mid		Med : High		Low : High	
	Median rho	p value sign-rank rho > 0	Median rho	p value sign-rank rho > 0	Median rho	p value sign-rank rho > 0
Monkey U	0.06	7.39×10^{-5}	0.112	1.4×10^{-8}	0.0065	0.27
Monkey V	.014	6.75×10^{-11}	0.10	1.3×10^{-6}	0.08	7.49×10^{-4}
Day-to-day bid 'coherence'						
	Low : Mid		Med: High		Low: High	
	Rho	p value Pearson	Rho	p value Pearson	Rho	p value Pearson
Monkey U	0.50	2.8×10^{-11}	0.51	8.0×10^{-12}	0.21	0.0094
Monkey V	0.54	8.5×10^{-12}	0.50	4.6×10^{-10}	0.23	0.0064

Supplementary Table 1. In the top rows labelled monkey U and monkey V, the median rho value for correlations between bids is provided along with the p-values for Wilcoxon sign-rank test for correlations between low and mid, mid and high, and low and high bids for each session. It is important to note that this provides a lower limit estimate of bid coherence as different reward magnitudes were presented in pseudorandom order on separate trials. The bottom rows show rho values and p-values for correlations of the average bids from day to day for each reward magnitude comparison (i.e., low to mid, mid to high, and low to high).
