# Supplementary material for: Dopamine signals encode internally determined subjective value regardless of externally indicated reward attributes
Source: bioRxiv. 2023 Jan 20:2023.01.20.524896. Preprint. [Version 1] doi: 10.1101/2023.01.20.524896 (PMC9882283; doi:10.1101/2023.01.20.524896)
Supplement: Supplementary file 1 [file NIHPP2023.01.20.524896v1-supplement-1.pdf]

SUPPLEMENTARY FIGURES

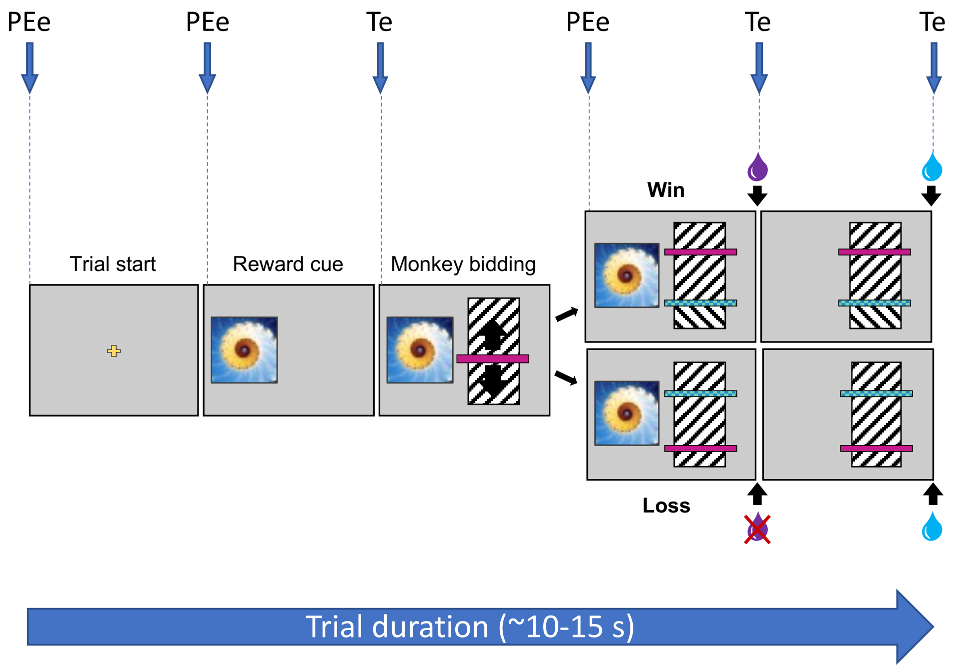

Figure S1. Task events. The analyzed relevant task events are shown as Te; Prediction Error events are denoted as PEe.

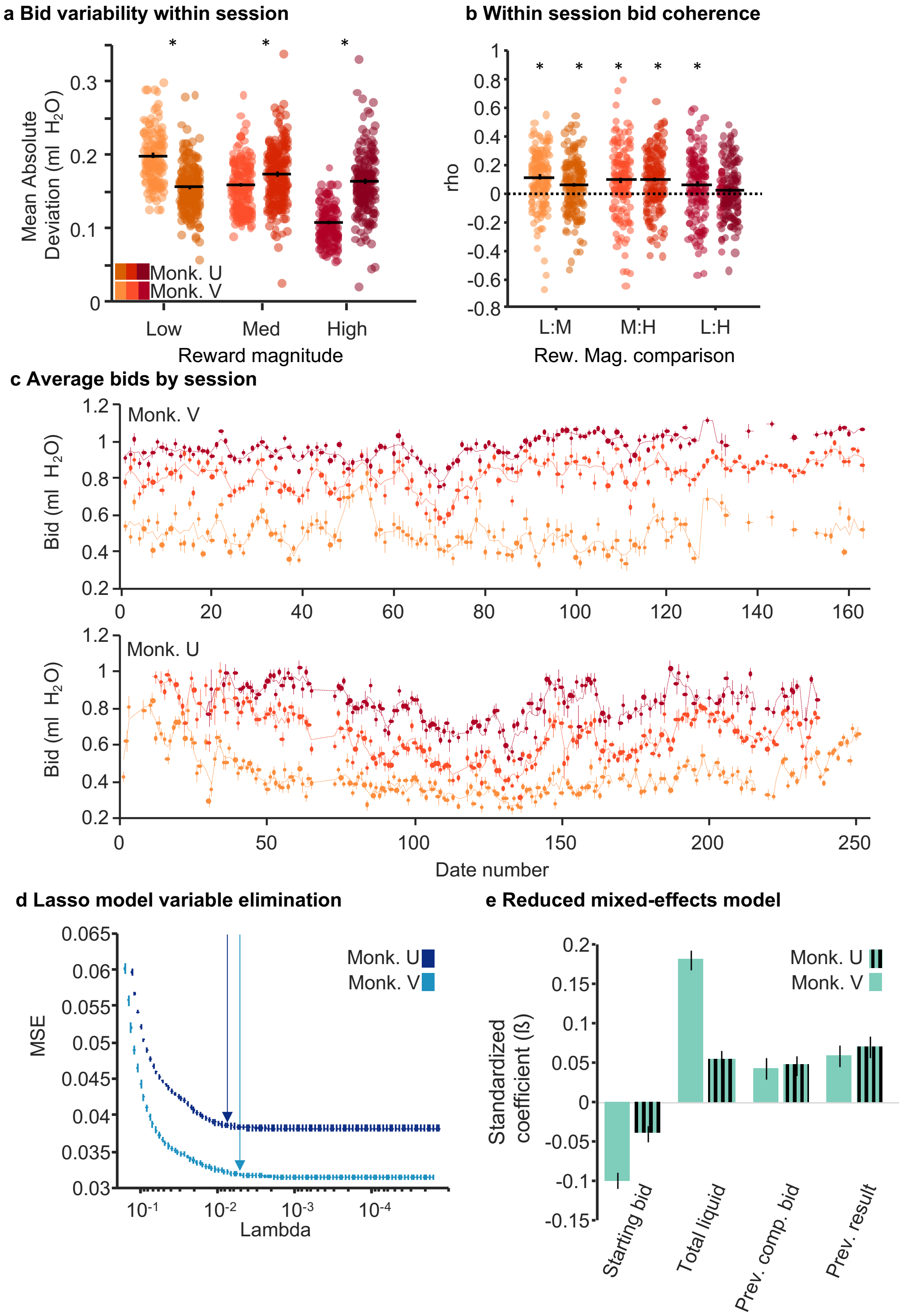

Figure S2. Bid characteristics. a, Bid variability within session. b, Bid coherence within session. c, Bid coherence between sessions. d, Cross-validated mean squared errors (MSE) for lasso regression model. A lasso model was used to eliminate task-irrelevant variables. The arrows indicate the lambda value that is one standard error above the minimum cross-validated error. This value was used as a cutoff; variables from this model were used in the mixed-effects model in figure 1 and in panel e. e, Mixed effects model controlling for reward magnitude. Mixed effects model identical to that in figure 1 with reward magnitude was included as a random effect. The model shows the contribution of relevant task variables to bid behavior independent of reward magnitude.

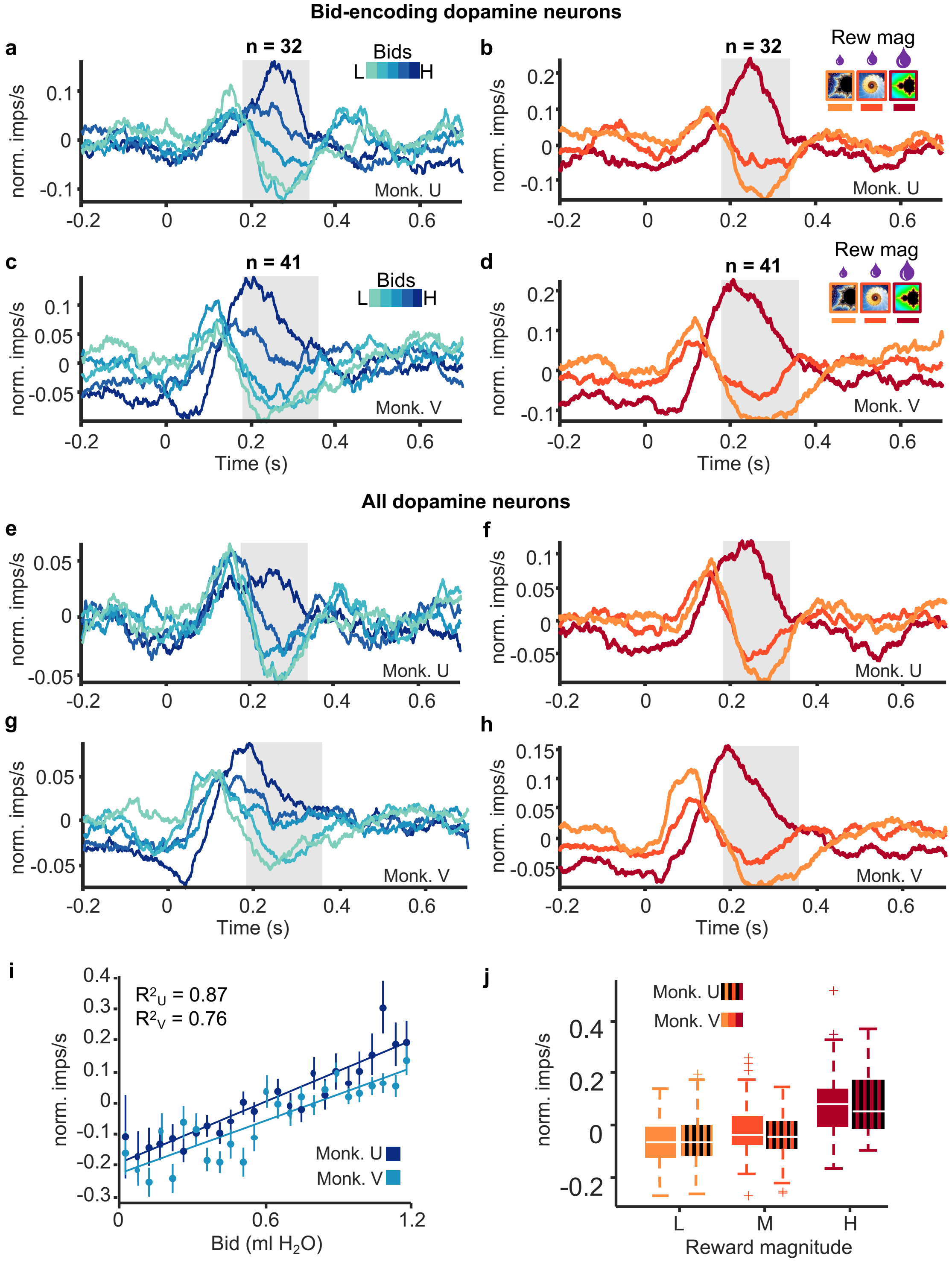

Figure S3. Bid-encoding and population responses. a-h, Average normalized dopamine responses for fifths of the bid-space for bid-encoding dopamine neurons (panels a-d) and for all dopamine neurons (panels e-h). i, Regression of normalized responses against bids for all dopamine neurons. Monkey U: $R^2 = 0.87$, $p = 1.03 \times 10^{-11}$, n = 32 neurons; Monkey V: $R^2 = 0.75$, $p = 1.64 \times 10^{-8}$, n = 41 neurons. j, Responses for all dopamine neurons grouped by reward magnitude. Kruskal-Wallis Test; Monkey U: $\chi^2 = 85.63$, $p = 2.55 \times 10^{-19}$, n = 123 neurons; Monkey V: $\chi^2 = 70.42$, $p = 5.11 \times 10^{-16}$, n = 145 neurons.

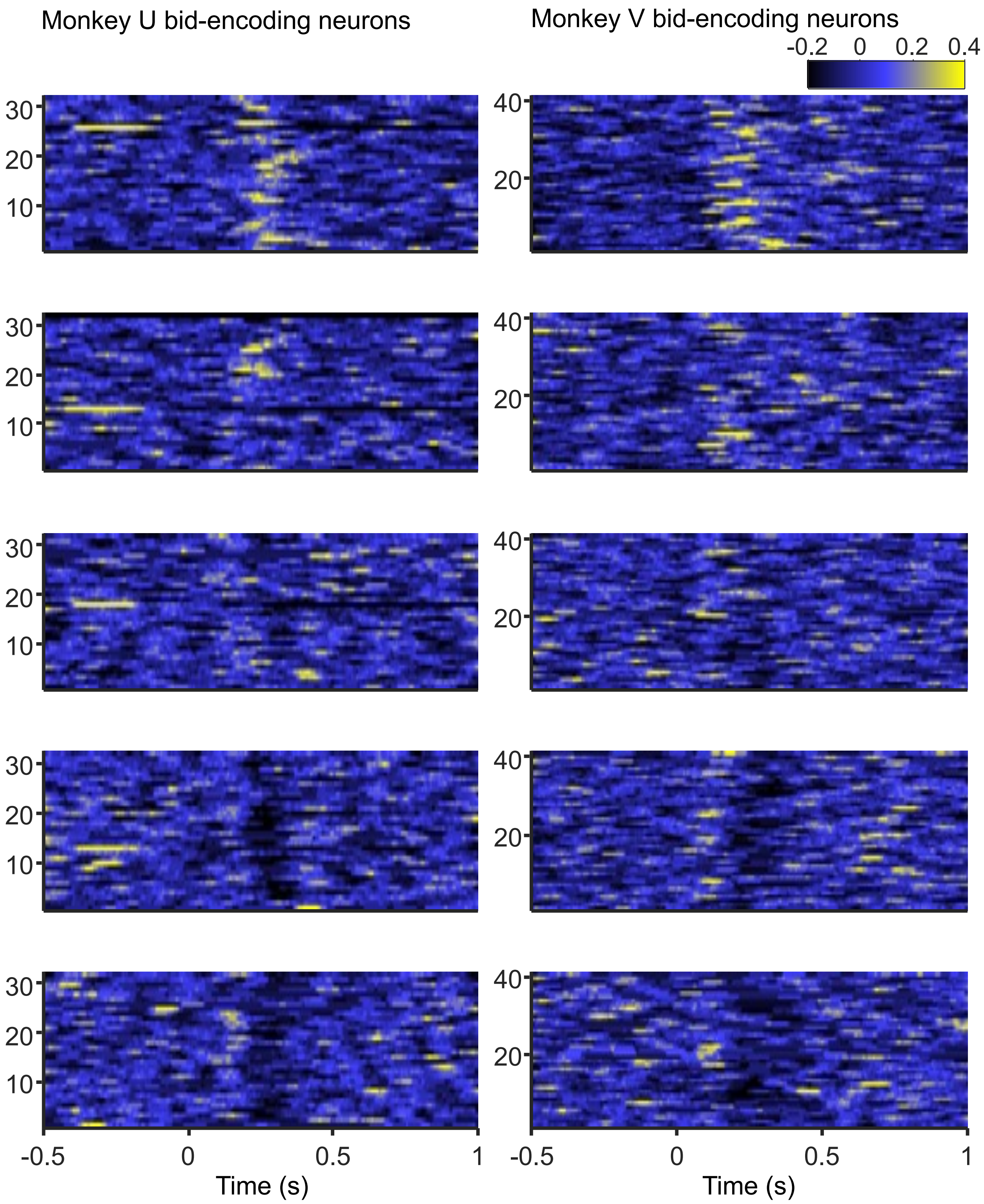

Figure S4. Average traces for each bid-encoding dopamine neuron for fifths of the bid-space. Average responses from the lowest fifth are shown at the bottom to the highest fifth at the top. Monkey U is on the left (n = 32), Monkey V is on the right (n = 41).

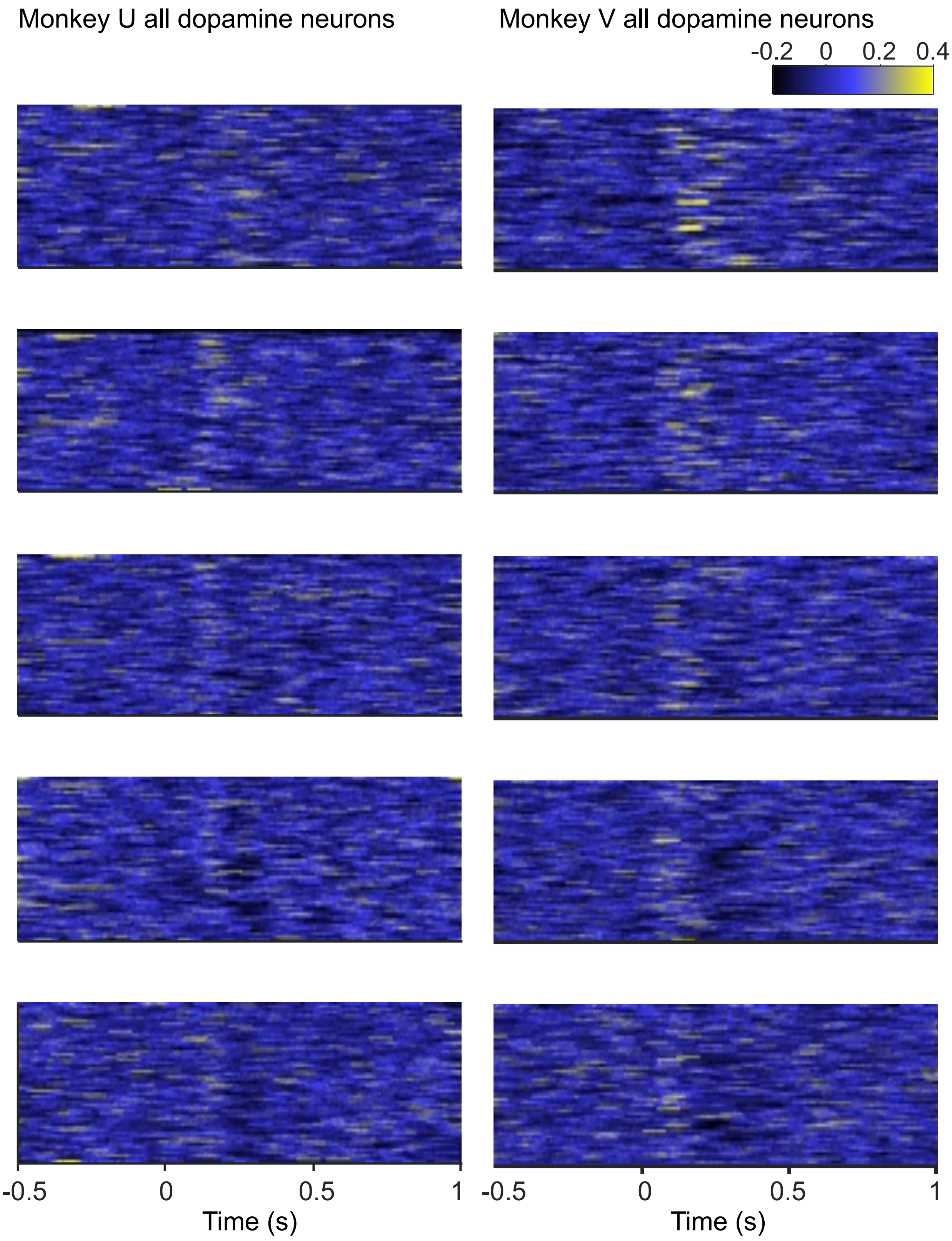

Figure S5. Average traces for all dopamine neurons for all five fifths of the bid-space. Average responses from the lowest fifth are shown at the bottom to the highest fifth at the top. Monkey U is on the left (n = 123 neurons), Monkey V is on the right (n = 145).

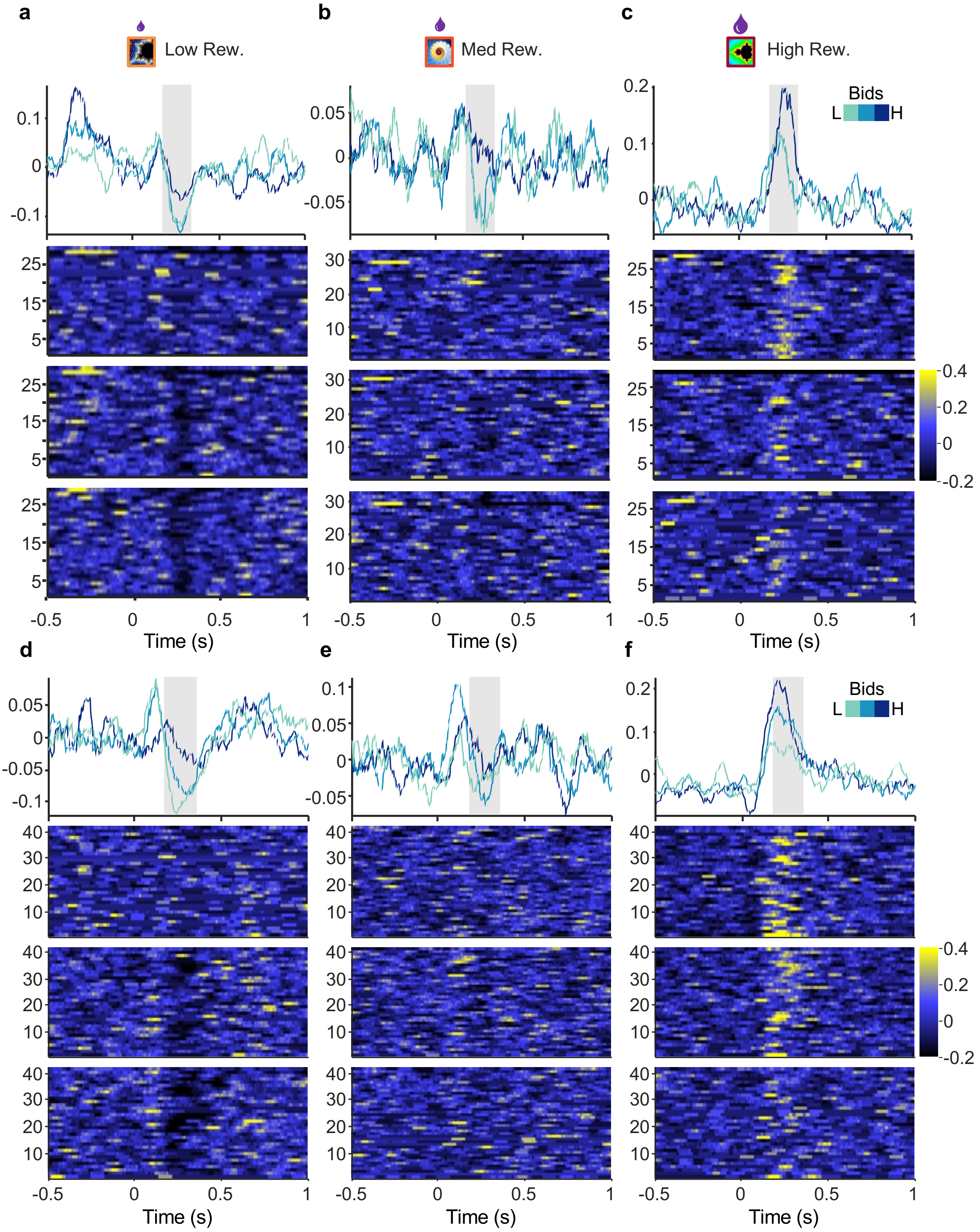

Figure S6. Average traces for each bid-encoding dopamine neuron for thirds of the bid-space within each reward magnitude (a, low; b, mid; c, high) for Monkey U (d-e; n = 32) and Monkey V (a-c; n = 41) . In each panel, responses from the highest, middle and lowest third are shown from top to bottom within each reward magnitude.

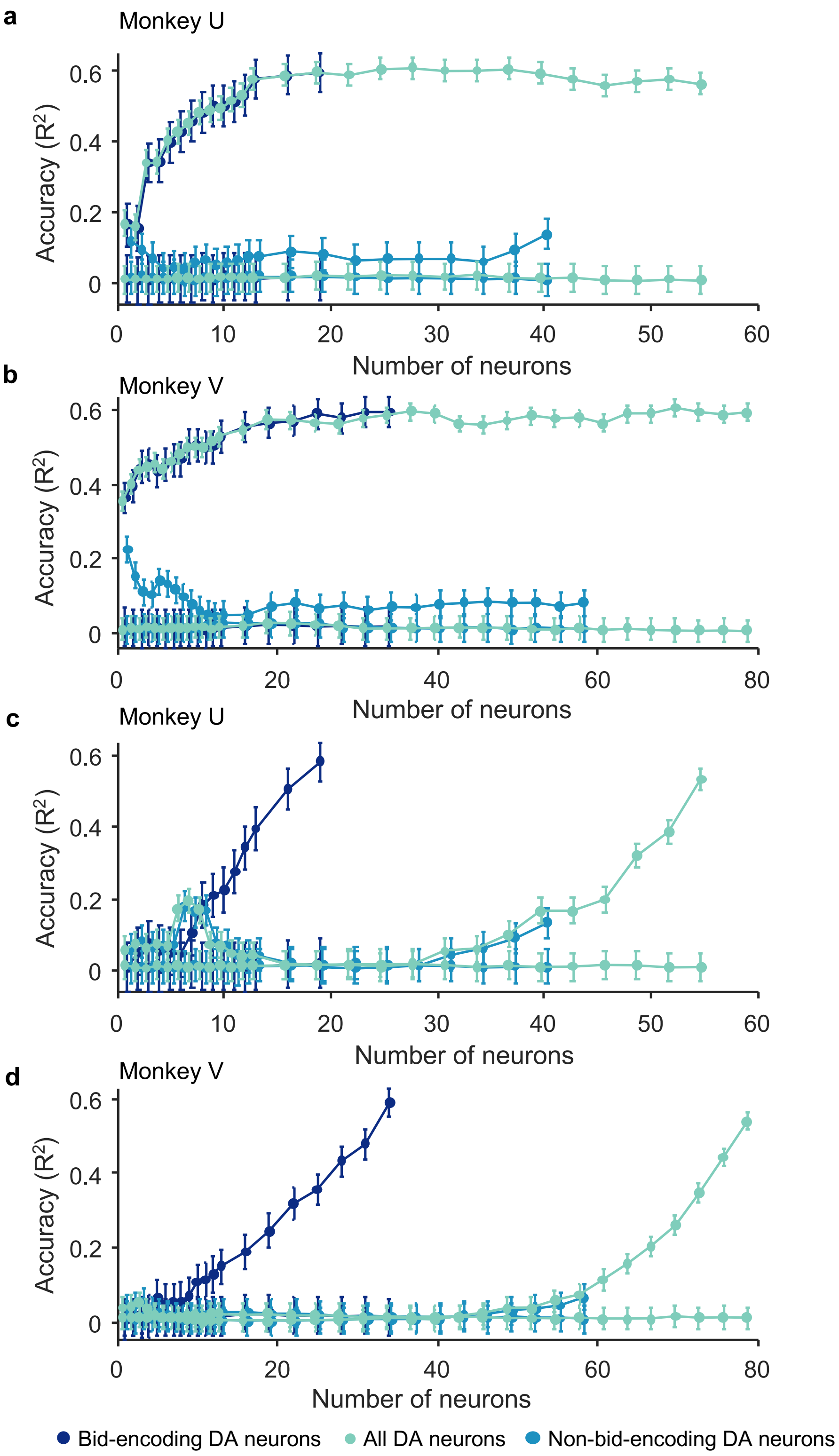

Figure S7. Results from analysis by Support Vector Regression (SVR) for the two monkeys. In contrast to figure 5, we added neurons from highest to lowest explained variance (derived from regression of individual neuron responses on bids) (a, b) and in reverse order (lowest to highest; c and d).

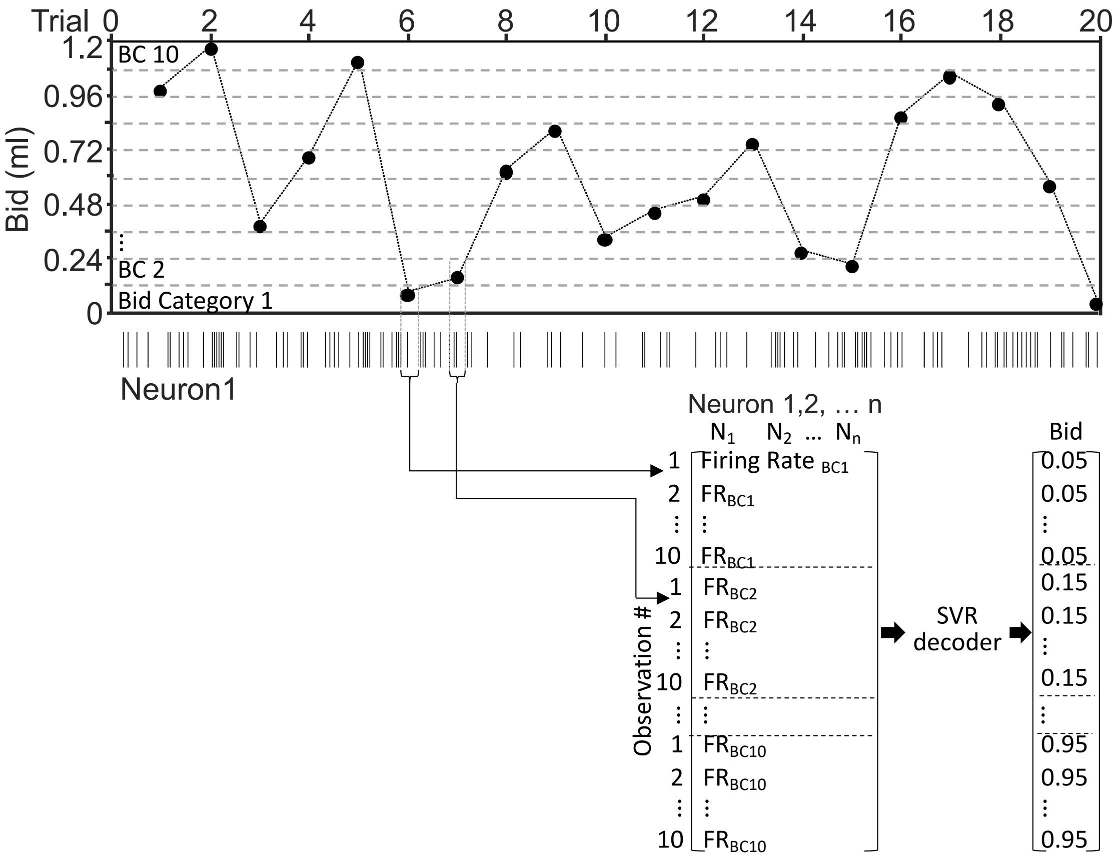

Figure S8. Design of the Support Vector Regression (SVR) model. Responses from 10 bid categories (BC; bid-space split into tenths) were added to the analysis such that each category was equally sampled. The bid-space was split into tenths to increase the sampling per bid category. The SVR was trained on these responses (see Methods).

SUPPLEMENTARY TABLE S1

Rho values and p values for time series bid ‘coherence’ (Pearson correlation).

Trial-to-trial bid ‘coherence’						
	Low : Mid		Med : High		Low : High	
	Median rho	p value sign-rank rho > 0	Median rho	p value sign-rank rho > 0	Median rho	p value sign-rank rho > 0
Monkey U	0.06	7.39×10^{-5}	0.112	1.4×10^{-8}	0.0065	0.27
Monkey V	.014	6.75×10^{-11}	0.10	1.3×10^{-6}	0.08	7.49×10^{-4}
Day-to-day bid ‘coherence’						
	Low : Mid		Med: High		Low: High	
	Rho	p value Pearson	Rho	p value Pearson	Rho	p value Pearson
Monkey U	0.50	2.8×10^{-11}	0.51	8.0×10^{-12}	0.21	0.0094
Monkey V	0.54	8.5×10^{-12}	0.50	4.6×10^{-10}	0.23	0.0064
